# The Anatomy of Inference: Generative Models and Brain Structure

**DOI:** 10.3389/fncom.2018.00090

**Published:** 2018-11-13

**Authors:** Thomas Parr, Karl J. Friston

**Affiliations:** Wellcome Centre for Human Neuroimaging, Institute of Neurology, University College London, London, United Kingdom

**Keywords:** Bayesian, neuroanatomy, active inference, generative model, message passing, predictive processing

## Abstract

To infer the causes of its sensations, the brain must call on a generative (predictive) model. This necessitates passing local messages between populations of neurons to update beliefs about hidden variables in the world beyond its sensory samples. It also entails inferences about how we will act. Active inference is a principled framework that frames perception and action as approximate Bayesian inference. This has been successful in accounting for a wide range of physiological and behavioral phenomena. Recently, a process theory has emerged that attempts to relate inferences to their neurobiological substrates. In this paper, we review and develop the anatomical aspects of this process theory. We argue that the form of the generative models required for inference constrains the way in which brain regions connect to one another. Specifically, neuronal populations representing beliefs about a variable must receive input from populations representing the Markov blanket of that variable. We illustrate this idea in four different domains: perception, planning, attention, and movement. In doing so, we attempt to show how appealing to generative models enables us to account for anatomical brain architectures. Ultimately, committing to an anatomical theory of inference ensures we can form empirical hypotheses that can be tested using neuroimaging, neuropsychological, and electrophysiological experiments.

## Introduction

This paper is based upon the notion that brain function can be framed as Bayesian inference (Kersten et al., 2004; Knill and Pouget, 2004). Under this view, our brains possess an internal (generative) model of our environment that we use to predict sensory data, and to explain current data in terms of their causes (Friston et al., 2006). Another way of putting this is that we have prior beliefs about the state of the world, and beliefs about how this gives rise to sensations. On encountering data, we update our beliefs to form a posterior belief about the world. This has interesting implications for neuroanatomy. Specifically, for most generative models, it is possible to specify belief updating, evidence accumulation or inference, in terms of the passing of local messages between variables in the generative model (Wainwright and Jordan, 2008). In machine learning, this gives rise to efficient inference schemes; including predictive coding (Rao and Ballard, 1999), variational message passing (Winn and Bishop, 2005; Dauwels, 2007), belief propagation (Yedidia et al., 2005; Pearl, 2014), and expectation propagation (Minka, 2001). In neuroscience, local message passing provides a useful way to interpret synaptic communication. This implies that the dependencies between variables in a probabilistic generative model should be reflected in the anatomy of the brain.

The good regulator theorem (Conant and Ashby, 1970) provides a useful perspective on the form generative models must take. It states that any system capable of regulating its environment must be a model of that environment. This means the model used by the brain must be constrained by what it tries to regulate; i.e., the body and its surroundings. By appealing to the form of the real-world processes that generate sensory data, we can attempt to construct generative models like those that the brain uses, and to use the inferential message passing those models imply to interpret known neuroanatomy. To do so, we need to understand the relationship between models and messages.

The key notion that underwrites this is the Markov blanket (Pearl, 2014)—a statistical boundary that renders one set of variables conditionally independent from another. Importantly, if we know everything about a variable's Markov blanket, knowledge about things outside the blanket becomes uninformative about things inside the blanket, and vice versa. For example, if we know the state of the surface of an object, the outside world offers no useful information about its interior. If we knew everything about the present, the past would add nothing to our predictions about the future. The concept of a Markov blanket is central to recent formulations of self-organization (Friston, 2013; Kirchhoff et al., 2018) and, in the present context, for anatomy. Here, the conditional dependency structure is important, as it means a population of neurons representing a given variable only need receive connections from those populations representing its Markov blanket.

Throughout, we will see that anatomy and generative models offer constraints upon one another that limit the space of plausible brain architectures. Ensuring mutual and internal consistency in these domains represents the kind of conceptual analysis necessary to form meaningful hypotheses in neuroscience (Nachev and Hacker, 2014). This is supported by the complete class theorems (Wald, 1947; Daunizeau et al., 2010) that ensure the validity of treating neural computations as Bayes optimal, and allow us to frame questions in terms of the generative models used for inference, and the physical (biological) substrates of these inferences.

This is not the first attempt to map inferential computations to the anatomy of the brain, and builds upon several existing accounts of neuroanatomy in terms of predictive coding architectures (Bastos et al., 2012; Shipp, 2016). The novel aspects of this article come from recent theoretical advances that address categorical inferences (Friston et al., 2012b, 2015), planning (Attias, 2003; Botvinick and Toussaint, 2012; Kaplan and Friston, 2018), and the inferences that underwrite the activity of the ascending neuromodulatory systems (Yu and Dayan, 2005; Friston et al., 2014; Parr and Friston, 2017c). In addition, our focus is upon the form of generative models and their constituent Markov blankets, while previous accounts have often focused upon the anatomy implied by specific inferential procedures. The ideas we present here transcend specific variational inference schemes and, for this reason, we avoid committing to a particular scheme in this paper. This is to emphasize that the conditional independencies in the generative model are the key constraints over anatomy.

The organization of this review is as follows. First, we overview the notion of a generative model and introduce some of the general principles that will be necessary for understanding the rest of the paper. Following this, we try to ground these abstract ideas by illustrating their implications in the domains of perceptual inference, planning, neuromodulation, and movement. We conclude by considering some specific and testable hypotheses.

## Generative models and markov blankets

**Figure 1:**
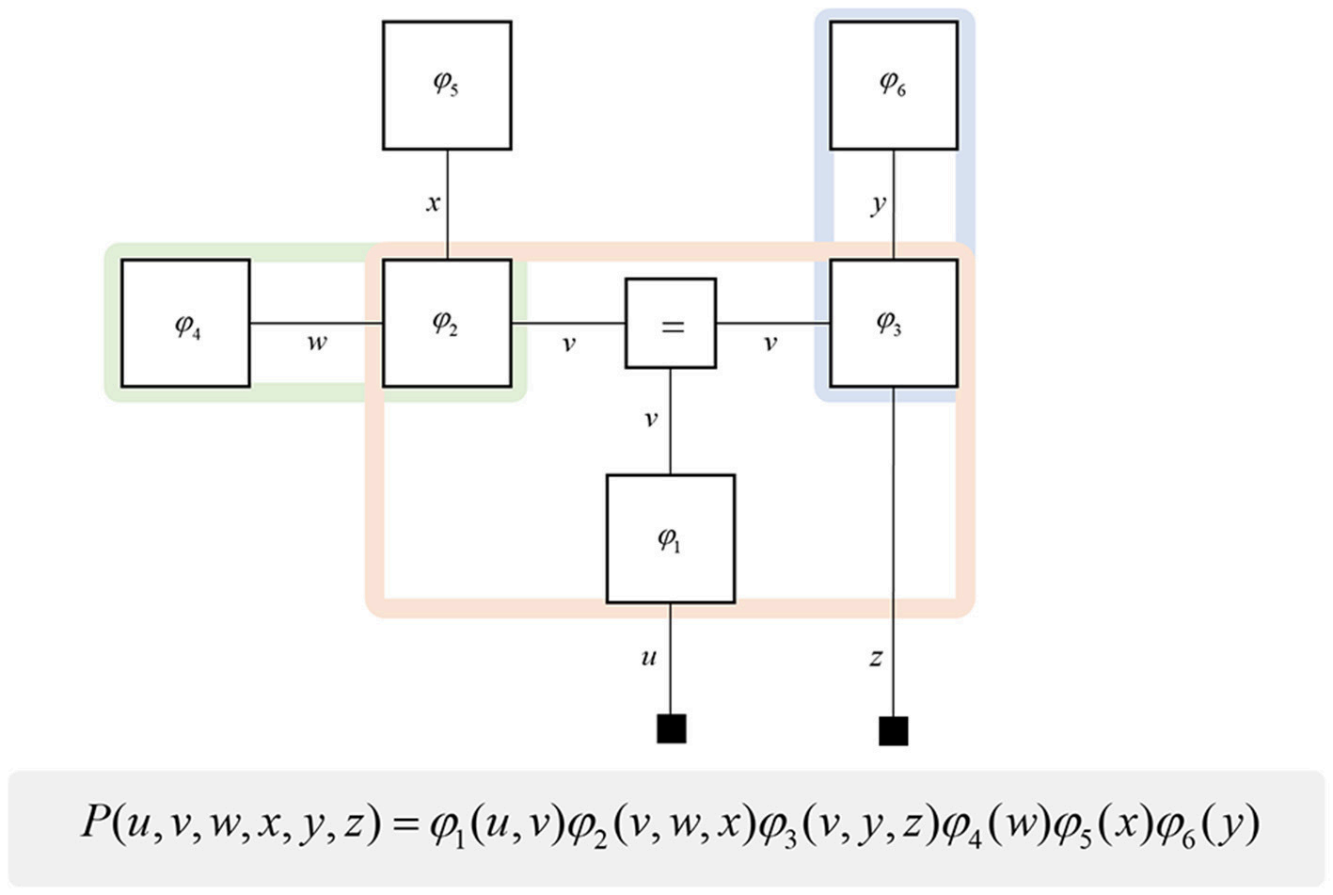
Forney factor graphs. The graphical model in this figure represents the (arbitrary) probability distribution shown below. Crucially, this distribution can be represented as the product of factors (ϕ) that represent prior and conditional distributions. By assigning each factor a square node, and connecting those factors that share random variables, we construct a graphical representation of the joint probability distribution. The “ = ” node enforces equality on all edges (lines) that connect to it. Small black squares represent observable data. This figure additionally illustrates a simple method for determining the Markov blanket of a variable (or set of variables). By drawing a line around all of the factor nodes connected to a variable, we find that the edges we intersect represent all of the constituents of the Markov blanket. For example, the green line shows that the blanket of *w* comprises *x* and *v*. The pink line shows the Markov blanket of *v*, which contains *u, w, x, y*, and *z*. The blue line indicates that *v* and *z* make up the blanket of *y*.

A generative model is a probabilistic description of how a given type of data might have been generated. It expresses prior beliefs about unobserved hidden states (or latent variables), the probabilistic dependencies between these states, and a likelihood that maps hidden states (i.e., causes) to sensory data (i.e., consequences). Such models can be used to predict new sensory data, and to infer the hidden states that could have caused observed data (Beal, 2003). While we rely upon several formal, mathematical, concepts in this paper, most of these formalisms can be expressed clearly through graphical models (Pearl, 1998). Inspired by recent papers that express electrical networks (Vontobel and Loeliger, 2003), analytical physics (Vontobel, 2011), and Quantum mechanics (Loeliger and Vontobel, 2017) using factor graphs, we adopt the same formalism to address computational neuroanatomy. Specifically, we use Forney-style factor graphs (Forney, 2001; Loeliger, 2004) as illustrated in Figure 1. This graphical notation provides a way to visualize any function that, like a probability distribution, may be decomposed into a product of factors. We use this notation for the generative models we will use in this paper. Each of these factors is plotted as a square node, and these are connected by an “edge” (line) if they are both functions of the same random variable. These graphs have previously been applied in the life sciences; notably, in theoretical neurobiology (de Vries and Friston, 2017; Friston et al., 2017a), and in biomedical engineering (Laar and Vries, 2016). One of the key advantages of these graphs is that they make the Markov blankets of each variable visually obvious.

A common rhetoric for describing directed causal relationships is that “parent” variables cause “child” variables. Using these terms, a Markov blanket for a given variable may be thought of as its parents, children, and the parents of its children (Pearl, 2014). While this is a simple rule to follow, it becomes even easier to identify blankets when adopting a factor graph formalism. This is because the constituents of a Markov blanket are the set of variables that share factors with those variables insulated by the blanket. Figure 1 illustrates a procedure that identifies all the components of the Markov blanket associated with a random variable, simply by drawing a line around the factors it participates in. Anatomically, these blanket components should correspond to the neuronal populations that project to that population housing representations of the original variable.

**Figure 2:**
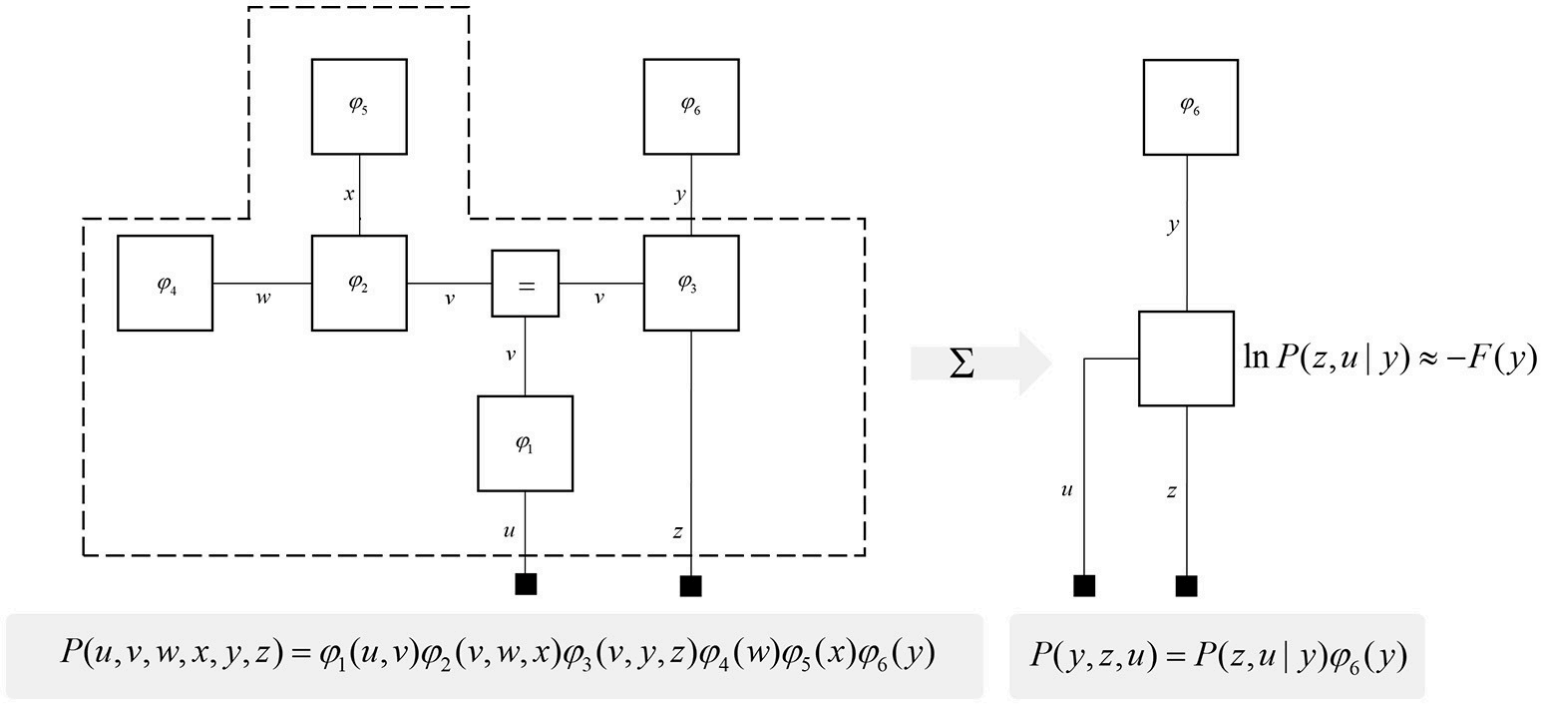
Partition functions and free energy. This schematic illustrates a useful operation known as “closing the box” or taking a partition function of part of a graph. By summing (or integrating) over all variables on edges within the dashed box, we can reduce this portion of the graph to a single factor that plays the part of a (marginal) likelihood. While it is not always feasible to perform the summation explicitly, we can approximate the marginal likelihood with a negative free energy. This affords an efficient method for evaluating subregions of the graph. Taking the partition function, or computing the free energy, for the whole graph allows us to evaluate the evidence sensory data affords the generative model.

One further concept that will be useful in what follows is the idea of “closing a box” (Loeliger, 2004) or finding the partition function (Forney and Vontobel, 2011) of part of the graph. This simply means summing (or integrating) over all of the variables represented by edges within a subgraph. Figure 2 demonstrates this idea by taking the graph of Figure 1 and converting it to a simpler graph by summing over all variables within a dashed box. For some generative models, this summation (or integration) may not be computationally or analytically feasible. However, we can approximate partition functions using free energy functionals (Dayan et al., 1995; Beal, 2003), as indicated in Figure 2. This becomes very important in active inference, which expresses brain function in terms of a principle of least action, that tries to minimize free energy over time (Friston et al., 2010). This is equivalent to pursuing behavior that gives rise to data consistent with the partition function of the brain's generative model; a process sometimes referred to as “self-evidencing” (Hohwy, 2016). This appeals to evidence in the technical sense (the probability that data could have been generated by a given model), which can be expressed as a partition function bounded by a free energy. A self-evidencing system is then one that acts to maximize the evidence for its model of its environment.

In the following sections, we will unpack the idea of a generative model and its constituent Markov blankets in several domains. Before doing so, it is worth emphasizing the domain generality of this sort of approach. The ideas here have been applied, explicitly or implicitly, across applications as diverse as agency (Friston et al., 2012b), simple forms of pictographic language (Friston et al., 2017c), and interpersonal interactions (Moutoussis et al., 2014). Ultimately, all of these rely upon the idea of the passing of local messages across graphs that represent generative models. The differences in each domain depend upon the specific form of the underlying generative model—but, crucially, not the principles of message passing and implicit functional architectures. In other words, the tenets of belief updating covered in this review should, in principle, apply to any perceptual or cognitive domain and their associated neuronal systems. Furthermore, the generative models illustrated below do not exist in isolation. Rather, it is the association between each part of the brain's generative model that facilitates complex behaviors requiring interplay between perception and action.

## Perceptual inference

### Dynamic generative models

**Figure 3:**
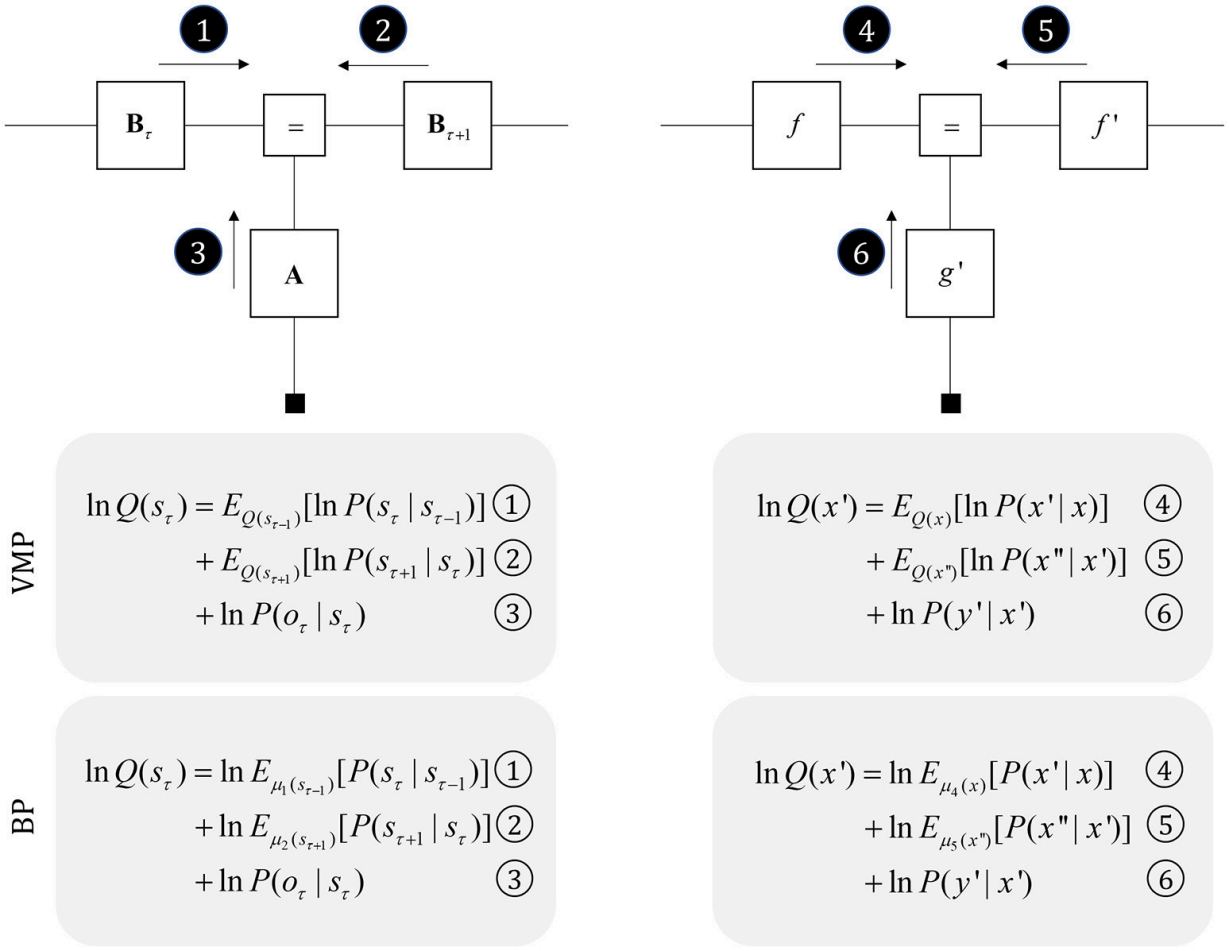
Perception as inference. This figure shows two generative models that describe hidden state trajectories, and the data they generate. On the left, we show evolution of discrete states (*s*), represented as the “edges” (lines) connecting the square nodes, with probabilistic transitions B from one state to the next. Each of these states gives rise to a discrete observation (*o*), as determined by a likelihood mapping A. On the right, we show the analogous factor graph for continuous dynamics, in which states are described in terms of their positions (*x*), velocities (*x*′), accelerations (*x*″), etc., coupled by flows (*f*) (rates of change) and give rise to continuous observations (*y*) determined by a likelihood mapping (*g*). Numbered black circles indicate the messages that would need to be passed to infer the current state (left) or velocity (right). The equations below show how these could be combined to form a belief about these variables using two different inference schemes (variational message passing and belief propagation). That the same message passing architecture applies in both cases emphasizes the importance of the generative model and its Markov blankets. The precise form of these message passing schemes is unimportant from the perspective of this paper, but for technical details on variational message passing schemes, we refer readers to Winn and Bishop (2005), Dauwels (2007), and for belief propagation (Loeliger, 2004; Yedidia et al., 2005), and to the Appendix, which provides a brief outline of these schemes. Table 1 provides a short glossary for some of the mathematical notation used in this and subsequent figures.

To start, we consider some of the simplest generative models that capture useful features of the environment. Broadly, there are two important categories (Friston et al., 2017a): those that describe the evolution of variables in discrete time (Mirza et al., 2016), and those that describe continuous dynamics (Friston et al., 2011). Trajectories in discrete time can be characterized by a sequence of values over time. Assuming a Markovian system, the value at any position in the sequence depends upon that at the previous time. During each time step, the current state of the world gives rise to a sensory observation. These probabilistic dependencies are illustrated in the factor graph on the left of Figure 3. As the Markov blanket of the present state includes the proximal past, future, and sensory observations, we only need messages derived from these to infer the present (Beal, 2003).

**Table 1:** Glossary of mathematical notation.

**Notation**	**Name**	**Description**
*P*( )	Generative model	A set of probability distributions that make up a generative model
*Q*( )	Posterior beliefs	An approximation to the probability of a variable given observed data
*H*[ ]	Shannon entropy	Uncertainty (or dispersion) of a probability distribution
*E*[ ]	Expectation	Expected (or average) value of a variable
*D_*KL*_*[ || ]	KL-Divergence	Difference between two probability distributions

It is also possible to represent trajectories in continuous time using a sequence of numbers, but these no longer express states at each time step. Instead, we can represent the coefficients of a Taylor series expansion of the trajectory. These are the current position, velocity, acceleration, and subsequent temporal derivatives—sometimes referred to as “generalized coordinates of motion” (Friston et al., 2010). This formalism is a very general way to represent trajectories, and encompasses similar formulations used for control systems (Baltieri and Buckley, 2018). On the right of Figure 3, we illustrate that this representation takes the same graphical form as the discrete case. The Markov blanket of the velocity includes the position, acceleration, and the rate of change of the data. Messages from each of these, under certain assumptions (Friston et al., 2007), take the form of squared precision-weighted prediction errors. The gradients of these are the messages passed by predictive coding schemes (Rao and Ballard, 1999; Friston and Kiebel, 2009).

Generative models that evolve continuous time or discrete time likely coexist in the brain, mirroring the processes generating sensory data. While, at the level of sensory receptors, data arrive in continuous time, they may be generated in a sequential, categorical manner at a deeper level of hierarchical structure. For example, a continuous model may be necessary for low level auditory processing, but language processing depends upon being able to infer discrete sequences of words (which may themselves make up discrete phrases or sentences).

### Neuronal architectures

**Figure 4:**
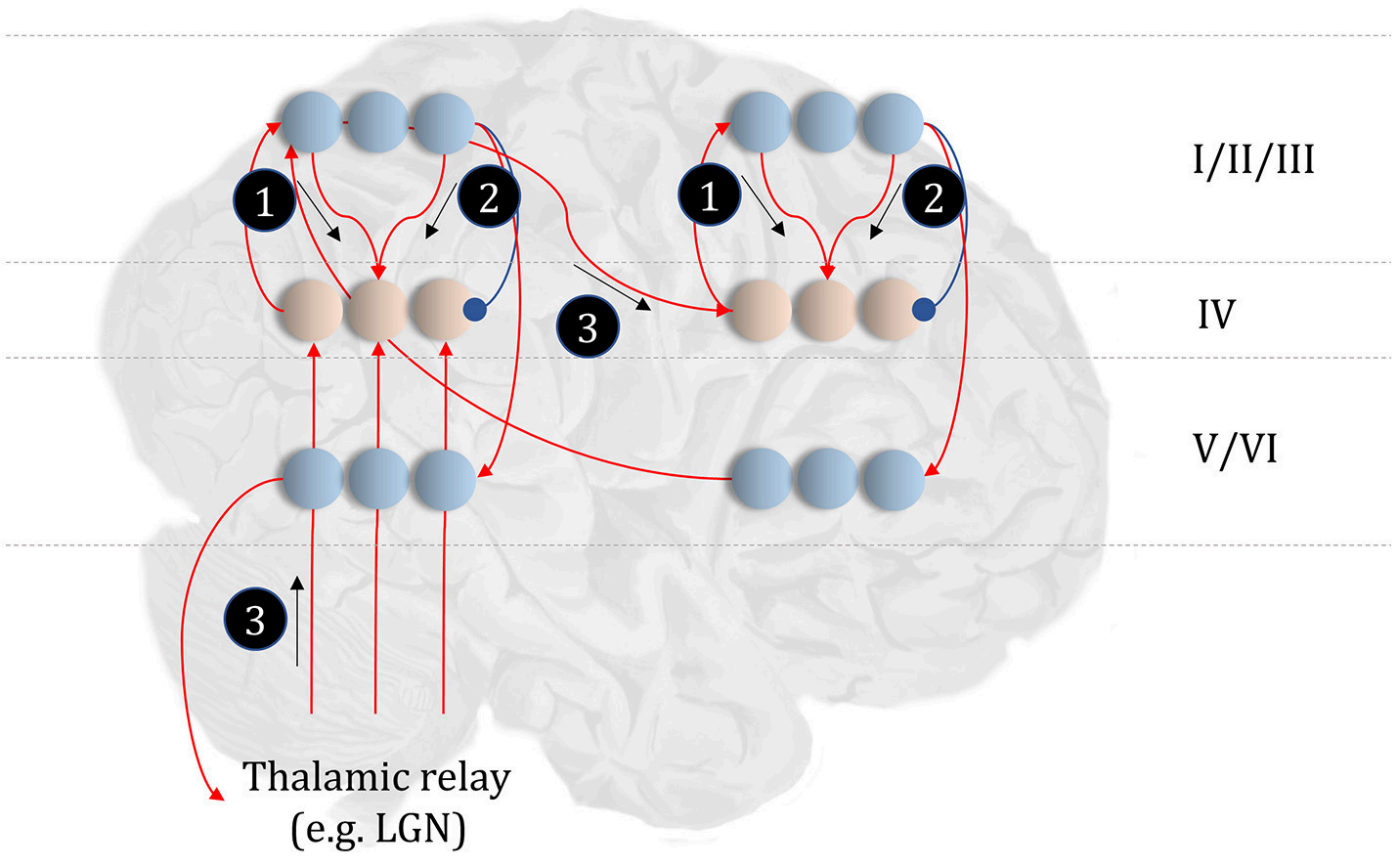
The anatomy of perceptual inference. The neuronal network illustrated in this figure could be used to perform inferences about the model of Figure 3. Neurons in cortical layer IV represent the spiny stellate cells that receive input from relay nuclei of the thalamus, and from lower cortical areas. The appropriate thalamic relay depends upon the system in question. In the context of the visual system, it is the lateral geniculate nucleus (LGN). In the somatosensory or auditory systems, it is the ventral posterior nucleus or the medial geniculate nucleus, respectively. Layer IV cells in this network signal prediction errors, computed by comparing the optimal estimate (obtained by combining the messages from its Markov blanket) with the current belief, represented in superficial cortical layers. Assuming a logarithmic code (as in Figure 3), this involves subtracting (blue connection) the current estimates of the sufficient statistics from the sum (red connections) of the incoming messages. The numbered circles indicate the same messages as in Figure 3. We could also represent messages 4, 5, and 6 in exactly the same way.

Before detailing the neuronal network that could perform these inferences, it is worth acknowledging the limitations of the generative model alone in trying to understand neuroanatomy at the microcircuit level. It may be that the brain makes use of auxiliary variables that, while in the service of inference, are not themselves sufficient statistics or messages. Probably the simplest example of this kind of variable is a prediction error, which quantifies the difference between the optimal solution and the current estimate of a continuous state (e.g., luminance contrast). In a biological setting, with inferences that play out in continuous time, gradient descents using prediction errors offer a plausible way to describe inferential dynamics (Rao and Ballard, 1999; Friston and Kiebel, 2009; Friston et al., 2017d). Figure 4 illustrates how we could represent the factor graph for discrete systems in neuronal network form, relating the messages highlighted in Figure 3 to axonal connections between populations of neurons. This represents input to prediction error cells in cortical layer IV that subtract current expectations, encoded in superficial layers, from the three incoming messages (which together represent the optimal expectation). These errors then drive changes in the superficial cells to update expectations (Miller, 2003; Shipp, 2016).

The move from the factor graph representation in Figure 3 to the neuronal network of Figure 4 proceeds as follows. First, each state (connecting line) in the factor graph is represented within a neuronal population in superficial levels of the network. The three units represented in Figure 4 correspond to beliefs about the state at three time-points. In the factor graph, these are the lines connected to the left **B**-factor (past state), the right **B**-factor (future state), and the line connecting the two (current state). We additionally include prediction error units in layer IV that relate to each of the belief states. As the prediction errors drive belief updating, they must receive the messages indicated in Figure 3, shown as connections from those populations from which the messages are derived in Figure 4. So far, we have accounted for layer IV and the superficial layers superimposed upon the posterior sensory cortices. The additional cortical column superimposed upon the frontal cortices replicates the same structure, but treats the states at the lower level as if they were sensory data.

Sensory input may come directly from sensory thalamic nuclei, or may come via sensory cortical areas (Thomson and Bannister, 2003). Both of these are shown in Figure 4, introducing the idea that the models in Figure 3 can form a repeating hierarchical pattern. This implies lower level hidden states may be generated from higher level states, where higher levels are defined as those that are further from sensory input. A consequence of this is bidirectional message passing between hierarchical levels (Friston et al., 2017c). This follows because a model that allows lower level states to be generated by higher level states mandates that each set of states sits in the Markov blanket of the other. An important feature of hierarchies in the brain is their temporal organization. As we ascend a cortical hierarchy, the temporal scale represented by each neuronal population generally increases (Hasson et al., 2008; Kiebel et al., 2008; Murray et al., 2014; Vidaurre et al., 2017). Those regions that sit near the top of these hierarchies are those associated with “delay period” activity or working memory (Funahashi et al., 1989; Goldman-Rakic, 1995); each defined by the persistence of a representation over a timescale that transcends that of stimulus presentation. To make the concept of a temporal hierarchy—or deep temporal models—more intuitive, consider the hierarchies inherent in reading: words are made up of letters, perceived over a fast time scale. Words themselves make up sentences, and paragraphs, each of which take longer to construct. Given the separation of timescales in real-world processes, like reading, the good regulator theorem (Conant and Ashby, 1970) implies generative models in the brain should adopt the same organization. As such, we could interpret a neuronal population at a higher level as encoding a short trajectory at the lower level, much as a sentence represents a short sequence of words. To illustrate this in Figure 4, we have only shown connections between the higher level cortical column and the first neuron in the sequence at the lower level. This is consistent with the generative models used to simulate reading (Friston et al., 2017c) and classic working memory tasks (Parr and Friston, 2017d) using active inference.

### Empirical constraints

This neuronal network illustrates a very important point. The architectures suggested by theoretical considerations must be constrained by our knowledge of real neuroanatomy (Douglas and Martin, 2004; Shipp, 2007). For example, sensory thalamic projections to the cortex, including those from the lateral geniculate nucleus, target the spiny stellate cells in (granular) layer IV of the cortex (Zeki and Shipp, 1988; Felleman and Van Essen, 1991; Callaway and Wiser, 2009). These cells project to more superficial layers, which themselves project to higher cortical regions. Connections from higher to lower regions of cortex (Bai et al., 2004), or from cortex to sensory thalamus (Olsen et al., 2012), arise from deep layers; notably layer VI (Thomson, 2010). To conform to this anatomy, the most obvious (but perhaps not only) solution is to assume that cells encoding the expectations are duplicated in the deep layers, illustrating the importance of mutual constraints between theory and known anatomy.

A similar constraint comes from neuropsychological research (Heinke and Humphreys, 2003; Testolin and Zorzi, 2016; Parr et al., 2018b). Not only should the networks we propose be internally consistent in both anatomical and theoretical domains, but they should give rise to similar deficits when disrupted; i.e., when the associated structures are lesioned. For example, if we were to remove message 3 (Figure 4) through a disconnection, or damage to the associated sensory organ, our prediction would be that internally generated influences (messages 1 and 2) would dominate perception. This is entirely consistent with conditions such as Charles Bonnet syndrome (Teunisse et al., 1996; Menon et al., 2003; Reichert et al., 2013), in which people with retinal damage experience complex visual hallucinations; something also associated with hypometabolism of early visual areas as observed with Lewy body dementia (Motohiro et al., 1987; Khundakar et al., 2016). Similarly, people with loss of proprioceptive or somatosensory input from an amputated limb can continue to experience percepts relating to absent body parts (Frith et al., 2000; De Ridder et al., 2014). These phantom phenomena are highly consistent with the theoretically derived architecture of Figure 4 (De Ridder et al., 2014). We will appeal to similar examples throughout, to illustrate the face validity of this anatomical process theory.

While the anatomy of Figure 4 might be suitable for describing trilaminar archicortical regions (Wesson and Wilson, 2011), such as the olfactory cortex and hippocampus, isocortical regions subdivide into six, histologically distinct, layers. To understand the need for additional inferential machinery, we note that perceptual inference is not a passive process. Sensory data depends to a large extent upon the orientation and position of mobile receptive epithelia (Parr and Friston, 2017a). This emphasizes the fact that an important class of latent variable is the set of hidden states over which we have control. These include the positions of body parts, and give rise to multiple data modalities (notably, visual, and proprioceptive). This provides an interesting perspective on the connections between frontal and posterior cortices, as the former houses representations of controllable variables, while the latter receives data about their sensory consequences (Shulman et al., 2009; Szczepanski et al., 2013; Limanowski and Blankenburg, 2018). Descending connections from frontal to parietal areas can then be thought of as predictions about the sensory input expected contingent upon a given action (Zimmermann and Lappe, 2016), endorsing an enactive perspective (Bruineberg et al., 2016; Kiverstein, 2018) on perceptual inference. In the context of the visual system, this implies visual space might be represented in terms of saccadic sensorimotor contingencies [i.e., “what I would see if I were to look there” (Parr and Friston, 2017a)]. The brain's ability to select future sensory data implies beliefs about the future, and about how it will choose to sample these data; i.e., planning.

## Planning

### Partition functions and (expected) free energy

**Figure 5:**
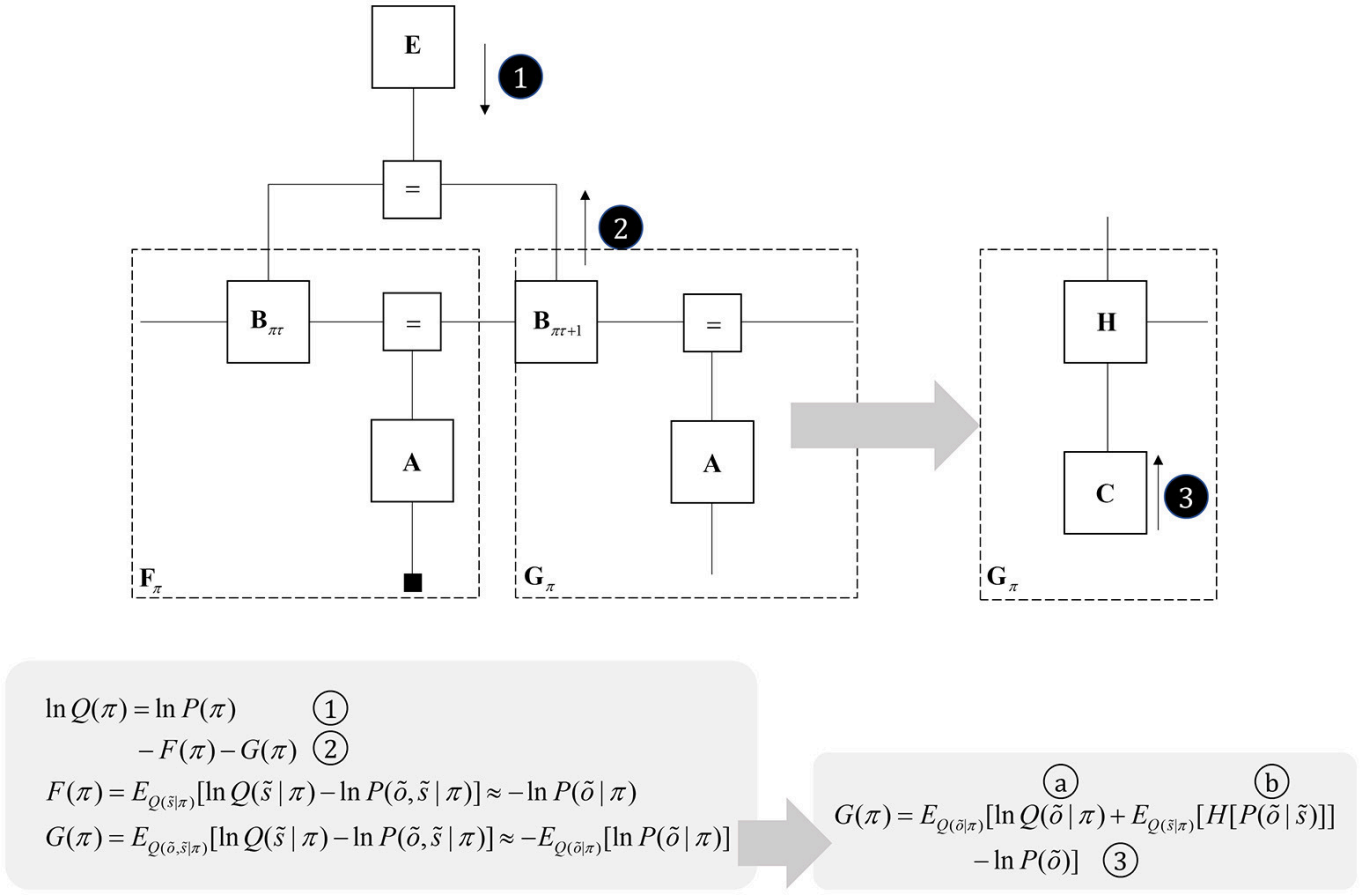
Planning as inference. This figure illustrates the use of partition functions to evaluate regions of the graph (see also Figure 2). Crucially, while we can approximate a partition functions based upon past data using a free energy functional, we do not yet have data from the future. This means we instead need an expected free energy, to approximate the partition function under posterior predictive beliefs. The panel below the graph illustrates how we can re-express the expected free energy such that we can represent this portion of the graph in terms of two new factors: a marginal belief about future outcomes and a likelihood that becomes an expected entropy after taking the expectation. As G depends upon beliefs about outcomes, but not upon the outcomes themselves, we can compute this prior to observing data. In some accounts of active inference, this is made explicit by treating C as a prior that connects to a factor G (that acts as if it were a likelihood generating policies from outcomes). The circled numbers and letters here are consistent with those in Figure 6. For technical accounts of these equations, please see Friston et al. (2017d), Parr and Friston (2018c).

One way to think about planning is that it represents the selection from several possible behavioral trajectories, or models of future action (Kaplan and Friston, 2018). This implies a set of models or policies that differ only in the state transitions they imply. We can represent this graphically by augmenting Figure 3 with a “policy” variable that represents which trajectory is in play. The edge related to this variable connects to the **B** factors, encoding transitions, and to an **E** factor, that represents a prior belief about which policy to engage in. To infer the appropriate behavioral policy, we can appeal to the idea of “closing the box” as in Figure 2, taking the partition function of the sequence of states, conditioned upon a policy, and their outcomes. As discussed in the section Generative models and Markov blankets partition functions are a way of summarizing part of a graphical model and may be approximated by a free energy functional. This suggests we can perform inference by passing messages in the subgraph within the dashed lines in Figure 5, computing posterior beliefs about the constituent variables conditioned upon a behavioral policy. These posteriors can then be used to calculate the free energy of each policy, providing evidence for or against each hypothesized trajectory. This treats planning as an inferential (Attias, 2003; Botvinick and Toussaint, 2012) model selection process.

Once we acknowledge the need for beliefs about the future, we run into a problem. By definition, sensory data from the future have not yet been collected, and we cannot compute their associated free energy. We can resolve this by using beliefs about the future to compute predictions about the sort of data expected under each policy. Averaging with respect to this “posterior predictive” density (probability distribution) allows us to compute an expected free energy (Figure 5) that approximates the partition function for future states and observations (Friston et al., 2015). This takes an interesting form when we use Bayes rule to re-express the generative model within the dashed box (gray arrow). Here, we have used the fact that the joint distribution over states and outcomes can be expressed either as the product of a prior and a likelihood, or as a posterior and a marginal distribution over the data (**C**). If we assume that the latter does not depend upon the policy, this acquires an important interpretation. As plans depend upon the negative expected free energy, those policies that give rise to data consistent with **C** become more likely to be selected. This allows us to think of this marginal distribution as encoding preferences or goals. Figure 5 illustrates this with a decomposition of the expected free energy (Friston et al., 2015) into a goal directed (message 3) and an uncertainty resolving (information gain) term (“a” and “b”). The consequence of this is that the best behavioral policies are those that balance exploitative (goal-directed) imperatives with explorative (information seeking) drives (Parr and Friston, 2017c).

Free energy approximations to model evidence (or expected model evidence) depend upon how closely beliefs (*Q*) approximate posterior distributions. This means that we must perform the message passing of Figures 3, 4 to ensure good approximations before using the free energy to adjudicate between policies that select new data as in Figure 5. We have previously argued (Friston et al., 2017d; Parr and Friston, 2018b) that these scheduling constraints may form the basis for theta rhythms in the brain, as this is the frequency at which we tend to sample the world around us (e.g., through saccadic eye movements). This implies that between actions we optimize posterior beliefs so that they can be used to compute free energy functionals to evaluate the next action.

### The basal ganglia

**Figure 6:**
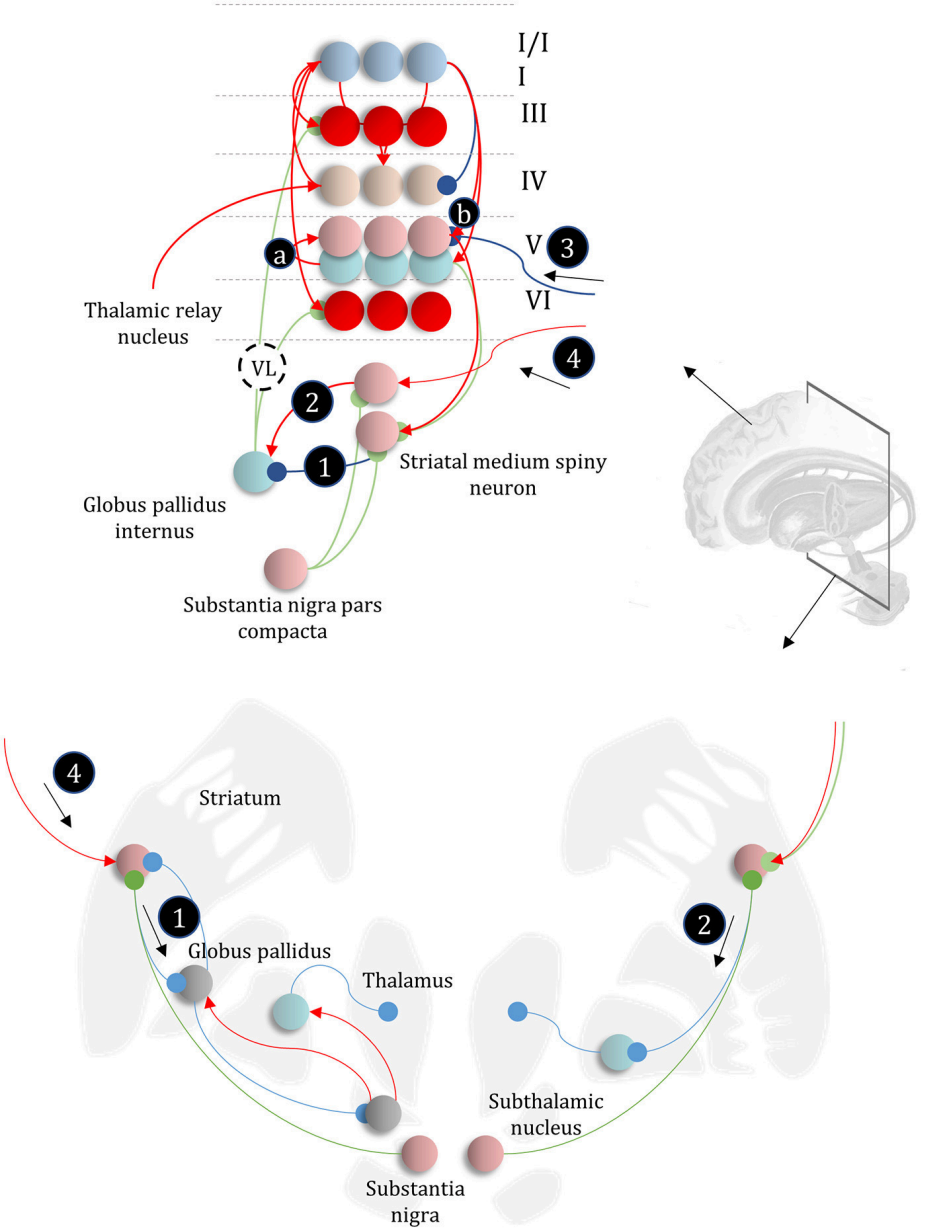
The basal ganglia. In the upper part of this figure, we show the same network as in Figure 4, but augmented such that it includes layer V cells encoding the gradients of the expected free energy and posterior predictive beliefs. These project to direct pathway medium spiny neurons and combine to give the expected free energy. This has a net inhibitory influence over the output nuclei (the globus pallidus internus and the substantia nigra pars reticulata), while the indirect pathway has a net excitatory effect. These are consistent with messages 2 and 3, respectively (the numbering is consistent with Figures 5, 7). Once the direct and indirect messages are combined at the globus pallidus internus, this projects via the thalamic fasciculus to the ventrolateral (VL) and ventral anterior nuclei of the thalamus. These modulate signals in the cortex, consistent with averaging beliefs about states under different policies, to compute average beliefs about the states (red neurons). Once we consider the hierarchical organization of this system (Figure 7), we need beliefs about preferences, derived from states at the higher level (message 3) combined with a posterior predictive belief (a) and an expected entropy term (b) to compute the gradient of the expected free energy. We additionally require a cortical input to the indirect pathway neurons, representing an empirical prior belief about policies (message 4—see Figure 7 for details). The coronal view of the basal ganglia, in the lower part of the figure, shows the connectivity of the direct (right) and indirect (left) pathways, to illustrate their consistency with the network above, but including the additional synapses that are not accounted for in the message passing. The substantia nigra pars compacta is included, and this modulates the weighting of messages 2 and 3. Please see the section below on Neuromodulation for details as to the emergence of dopaminergic phenomena from a generative model. In summary, the layers of the cortical microcircuit shown here represent beliefs about states under a given policy (I/II), beliefs about states averaged over policies (III), state prediction errors (IV), expected free energy gradients and predicted outcomes (V), and beliefs about states averaged over policies (VI).

The basal ganglia are a complex network of subcortical structures (Lanciego et al., 2012). They are engaged in a set of hierarchically organized cortico-subcortical loops, thought to underwrite planning and behavioral policy selection (Haber, 2003; Yin and Knowlton, 2006; Graybiel and Grafton, 2015; Jahanshahi et al., 2015). The anatomy of cortico-basal ganglia communication depends upon two distinct circuits, referred to as the direct and indirect pathways. Each has an opposing influence upon behavior, with the direct pathway facilitating, and the indirect suppressing, voluntary behaviors (Freeze et al., 2013). It is tempting to associate the two different messages (Figure 5) required to compute beliefs about policies with each of these pathways, as in Figure 6.

The striatum, consisting of the caudate and putamen, is the main input nucleus of the basal ganglia (Shipp, 2017). It is the origin of the direct and indirect pathways, associated with the initiation and inhibition of behavioral policies, respectively. Each pathway is associated with phenotypically distinct striatal medium spiny neurons. They are often characterized pharmacologically, with facilitatory D1-dopamine receptors predominating in direct pathway neurons, and suppressive D2-dopamine receptors on indirect pathway neurons (Smith et al., 1998); allowing dopamine to modulate the balance between these two pathways. However, the phenotypic differences extend beyond pharmacology to include anatomical connectivity, morphology, and electrophysiological properties (Gertler et al., 2008).

The differences between direct and indirect pathway medium spiny neurons are consistent with the form of the messages required to compute posterior beliefs about policies. While the direct pathway neurons have an inhibitory effect on the output nuclei of the basal ganglia, the indirect pathway has a net excitatory effect. The latter depends upon an additional GABAergic (inhibitory) synapse from the globus pallidus externus to the subthalamic nucleus (Jahanshahi et al., 2015), which has (excitatory) glutamatergic projections to the globus pallidus internus, converging with the direct pathway (Figure 6). Morphologically, D1-expressing neurons are well suited to combining input from many cortical areas to compute a free energy (or expected free energy) functional (Gertler et al., 2008). Compared to their D2-expressing counterparts, they have an extensive dendritic arbor, accompanied by a relatively high action potential threshold, suggesting their firing is highly context dependent, drawing from a wide range of cortical areas. Given the representation of posterior beliefs in the cortex (Figure 4), this suggests direct pathway medium spiny neurons are very well placed to compute the free energy expected under a given policy (i.e., message 2 in Figure 5).

**Figure 7:**
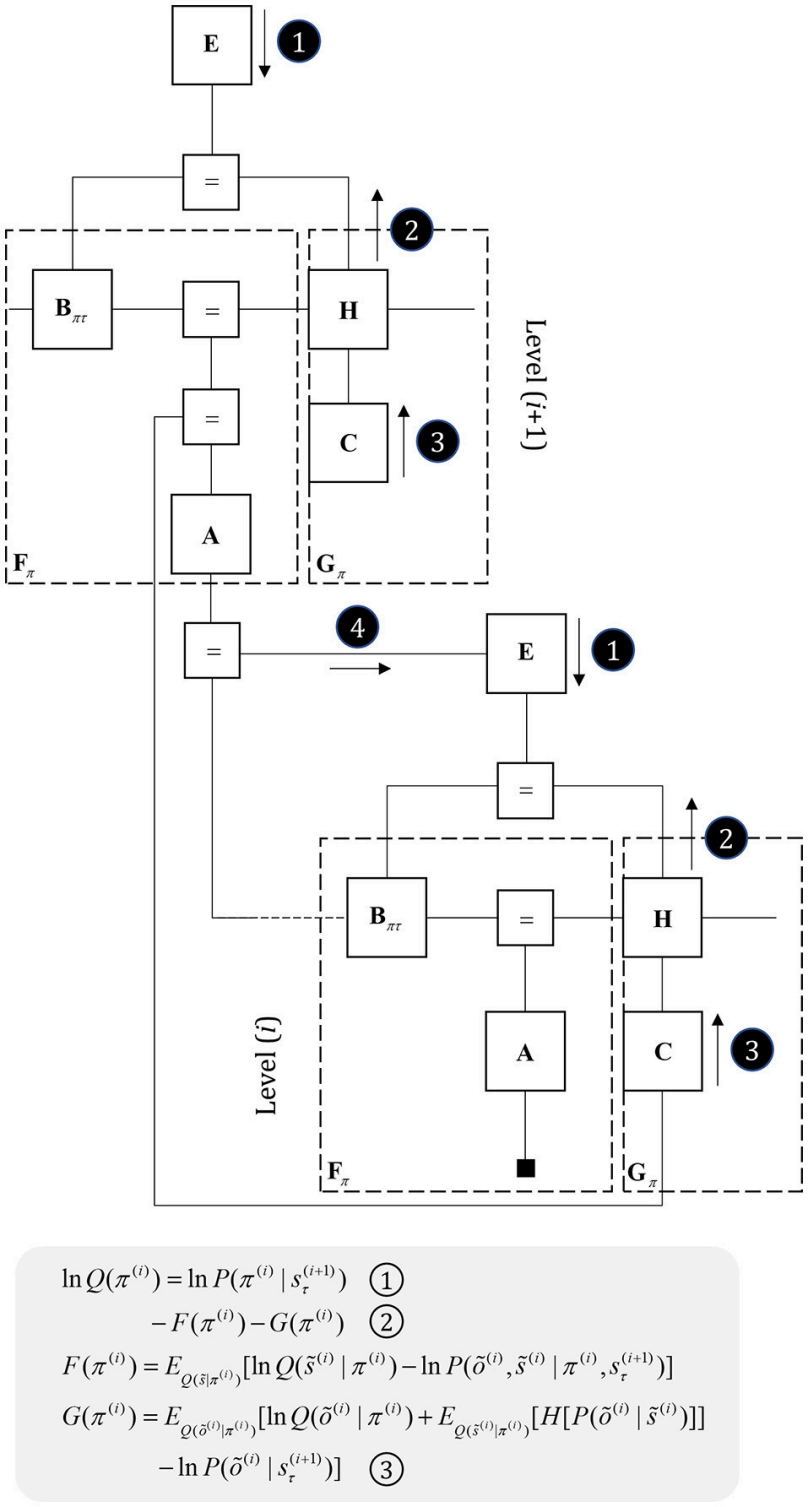
Hierarchical models. This figure illustrates the extension of Figure 5 to two hierarchical levels; although this pattern could be recursively extended to an arbitrary number of levels. There are three points at which the levels interact. The first is a mapping from the outcomes of the higher level to the initial states at the lower level (A to “ = ” to B). An example of this might be a mapping from a sentence level representation to the first word of that sentence. The second associates higher level outcomes with low level empirical priors over policies (A to “ = ” to E). Finally, we allow the low-level preferences to depend upon higher level states (“ = ” to C).

In contrast, indirect pathway striatal neurons have a smaller dendritic arbor (Gertler et al., 2008). This is consistent with message 1 in Figure 5, that does not depend upon cortically held beliefs (Parr and Friston, 2017b). The idea that the message from these neurons is independent of the cortex is clearly a step too far, as these cells do receive some cortical input. One way to resolve this is to note that the factor **E**, while playing the role of a prior, becomes an *empirical* prior^1^ once we move to a hierarchical network (Figure 7). This is because we can treat prior beliefs about policies as conditioned upon the outcomes at a higher level. In other words, habitual behaviors are dependent upon a slowly changing context. Interpreting cortical input to the indirect pathway as a descending signal from higher cortical regions is consistent with the difference in the distribution of cortical input to the direct vs. indirect pathways (Wall et al., 2013). The latter tends to receive more input from frontal regions, often thought to sit higher in cortical hierarchies (Felleman and Van Essen, 1991) than the sensory regions projecting to the direct pathway neurons. Endorsing a hierarchical aspect to basal ganglia function, the striatum reflects the hierarchical structure of the cortex in the connections it receives, its interactions with the midbrain, and the behaviors it modulates. For example, dorsolateral parts of the striatum receive dopaminergic input from the substantia nigra pars compacta and cortical input from sensorimotor cortices (Haber, 2003). In contrast, the ventral striatum receives dopaminergic input from the ventral tegmental area, and cortical input from limbic cortices.

### Cortico-subcortical networks

In addition to providing a computational hypothesis for basal ganglia function that formalizes the notion that they are engaged in planning (i.e., policy evaluation), we can now refine the cortical anatomy of Figure 4 to include the signals required to compute the expected free energy in the striatum (Friston et al., 2017c). In Figure 6, we show the addition of two cell populations in layer V. These represent posterior predictive beliefs about the sensory outcomes of a given policy, and the gradient of the expected free energy with respect to these beliefs. The latter is an auxiliary variable, like the prediction errors of Figure 4. It is computed based upon the **C** factor, the entropy^2^ of the likelihood (a) (weighted by beliefs about states), and posterior predictive beliefs about outcomes (b) (Friston et al., 2017a). Weighting the expected free energy gradients by these predicted outcomes allows us to compute the expected free energy associated with a given policy. The reason for restricting these cell populations to cortical layer V is that the cortical input to striatal medium spiny neurons arises almost exclusively from this layer (Shipp, 2007; Wall et al., 2013). There are several types of layer V pyramidal cell, two of which project to the striatum (Kim et al., 2015). One of these additionally projects to subcortical regions, including the superior colliculus, and we will return to this in the section on Movement. A further modification of the anatomy of Figure 4 is that we have included beliefs about states under a given policy (layers I and II), and beliefs about states averaged over all policies (layers III and VI) (FitzGerald et al., 2014). The latter are the sources of ascending and descending messages, and are computed by weighting conditional beliefs from superficial layers by beliefs about policies from the output nuclei of the basal ganglia (via the ventrolateral nucleus of the thalamus McFarland and Haber, 2002).

While the computational anatomy appears to be consistent with known basal ganglia circuitry, there are several outstanding questions that need resolution. The first of these is the number of synapses between the indirect pathway neurons and the output nuclei. The upper and lower parts of Figure 6 emphasize this, with the inclusion of intermediate synapses in the lower part. While the direct pathway involves a single synapse between the striatum and globus pallidus internus (or substantia nigra pars reticulata), the indirect pathway is trisynaptic. A single additional inhibitory synapse makes intuitive sense, as this converts a net inhibition to an excitation (consistent with message 2 in Figure 6). However, the additional excitatory synapse appears redundant. A plausible theory—that accounts for this—relies upon the timing of messages in each pathway (Nambu, 2004). This suggests that a short latency signal from the direct pathway disinhibits a select set of policies, based upon the highly specific contextual signals it receives from the cortex. This is followed by a much broader inhibition of *a priori* implausible policies by the slower indirect pathway. The timing of these signals is thought to contribute to a center-surround pattern of activity in the basal ganglia outputs which ensures precise posterior beliefs about policies. We could also argue that, if the indirect pathway receives slowly changing contextual input from higher cortical areas, it makes sense that its signals should play out over a longer time-span. The presence of recurrent connectivity within the indirect pathway, including arkypallidal neurons (Mallet et al., 2012) from the external globus pallidus to the striatum, reinforces this idea, as this could sustain these representations over a longer time.

A second question concerns the role of the hyperdirect pathway (Nambu et al., 2002), which provides a subthalamic input, and why it is necessary to have an additional cortical input. A plausible, if speculative, role for this pathway is in signaling when to implement each new policy, as these cortico-subthalamic axons arise from motor neuron collaterals (Giuffrida et al., 1985). Given the highly non-specific terminations of the hyperdirect pathway in the basal ganglia outputs, it seems more likely that this signals when an action has taken place, as opposed to contributing directly to policy selection. This is consistent with the theta cycle at which new actions (e.g., saccades) are performed (although the components of these actions may be much faster), and its correlates in the subthalamic nucleus during sensorimotor tasks (Zavala et al., 2017). The hyperdirect pathway may then use motor signals to entrain the pacemaker circuits associated with this nucleus (e.g., subthalamopallidal networks) that include neurons oscillating at a theta frequencies (Plenz and Kital, 1999). Speculatively, this could be an important part of the scheduling of the message passing involved in planning, compared to that for state inferences. Failures of scheduling may underwrite aspects of conditions like Parkinson's disease (Cagnan et al., 2015).

### Pathologies of the basal ganglia

Disorders of basal ganglia nuclei are well characterized. It is a useful test of the validity of the proposed anatomy to see whether deficits in these computational units are consistent with behaviors observed in neurological practice. Parkinson's disease is a common disorder in which degeneration of neurons in the substantia nigra pars compacta lead to dopamine deficits in the striatum (Albin et al., 1989). This leads to an akinetic rigid syndrome, characterized by a difficulty initiating movements (Clarke, 2007). If we interpret dopamine as weighting the balance of messages derived from expected free energy compared to empirical priors (Friston et al., 2014; FitzGerald et al., 2015; Schwartenbeck et al., 2015), the loss of dopamine could lead to excessive reliance on these slowly changing priors that do not take account of changes in context over the timescale necessary for movement. We have previously demonstrated behaviors that become increasingly random as the contribution from the expected free energy is down-weighted in the presence of flat priors (Parr and Friston, 2017d). Under the more realistic priors required for (for example) maintenance of postural stability (Dokka et al., 2010), it is easy to see how an excessive reliance upon these might lead to akinesia and rigidity. This suggests Parkinson's disease could be thought of as a syndrome of excessive reliance upon slowly changing beliefs at higher hierarchical levels to direct behavior (Jávor-Duray et al., 2017). Reliance upon higher levels might account for the bradykinesia and bradyphrenia of this disorder (Mayeux, 1987), as faster processes lose their influence over (motor and mental) behavior. In contrast, increasing dopamine levels might decrease the influence of higher levels, leading to shorter term planning and impulsive behaviors of the sort associated with pro-dopaminergic Parkinson's medications (Cools et al., 2003; Michele and Anna Rita, 2012).

An intriguing feature of Parkinson's disease is that, in certain contexts, patients can perform complex fluent motor behavior; e.g., cycling (Snijders and Bloem, 2010). This phenomenon, known as *kinesia paradoxa* (Banou, 2015), typically relies upon some form of cueing (Glickstein and Stein, 1991), signaling a behavioral context. The hierarchy of Figure 7 offers a framework in which we can try to understand this effect. While dopaminergic deficits limit the influence of message 2 on policy selection, there is another route by which sensory data can influence behavior. Although somewhat circuitous, messages may be passed up to the higher level, allowing inference about slowly changing hidden states. These then influence lower level policies via message 4. This indicates that understanding the structure of message passing architectures might afford opportunities for the design of rehabilitative therapies or medical devices (Ferrarin et al., 2004) that make use of alternative routes through the set of Markov blankets comprising a generative model.

While Parkinson's disease represents reduced direct pathway influences, there are other syndromes that occur if the indirect pathway is damaged. These provide support for the idea that the indirect pathway uses prior beliefs to prevent the performance of implausible behavioral policies. One such syndrome is hemiballismus, resulting from damage to the subthalamic nucleus (Hawley and Weiner, 2012). This syndrome is characterized by involuntary ballistic movements that the indirect pathway fails to suppress. This is consistent with a policy that has a relatively low expected free energy, despite being implausible according to healthy prior beliefs. Crucially, while disconnections in the indirect pathway lead to fast involuntary movements, reduced direct pathway influences lead to slowing of movements. The difference in time scales adds further weight to the hypothesis that indirect pathway signals derive from slower hierarchical levels.

### Other subcortical networks

The ideas in this section may be generalizable to other subcortical structures. Specifically, some nuclei of the amygdala resemble those of the basal ganglia, but appear to have a role in regulating autonomic, as opposed to skeletomotor, policies (Swanson and Petrovich, 1998; Kimmerly et al., 2005). The central and medial nuclei appear to be extensions of the striatum and may send and receive analogous messages, suggesting these nuclei compute the expected free energy of alternative autonomic policies. The output of these amygdala regions target the periaqueductal gray matter (Hopkins and Holstege, 1978; Bandler and Shipley, 1994) and hypothalamic regions (Petrovich et al., 2001) that regulate the balance between the sympathetic and parasympathetic nervous systems. This view of these structures is highly consistent with inferential accounts of autonomic regulation (Owens et al., 2018). That the same computational role associated with the basal ganglia generalizes to provide a hypothesis for the function of some amygdala nuclei suggests that similar explanations might hold for other subcortical structures. The generative model we have considered so far leads us to anticipate that any planning or decision-making process, whether in the domain of skeletomotor, autonomic, or mental (Metzinger, 2017; Limanowski and Friston, 2018) action, implies an anatomical network for the evaluation of expected partition functions, or free energies, for alternative courses of action.

## Neuromodulation

### Precision and attention

In addition to knowing which variables are causally related to which others, our brains must be able to infer how reliable these relationships are. In the previous section, we discussed the role of dopamine in modulating the indirect and direct pathways, but without specifying its role in the generative model. We have suggested that dopamine increases the influence of message 2 relative to message 1 (Figure 7); i.e., changes the weighting of priors and likelihoods. This implies it plays the role of a precision parameter. Precision quantifies the confidence, associated with a given probabilistic distribution. Associating a high precision with prior beliefs means that these are the dominant influence in forming a posterior belief. Lower precisions of prior beliefs favor other messages, including those derived from sensory data. We could then think of dopamine as encoding the imprecision of prior beliefs about policies or, as the precision of the partition functions used to evaluate the evidence for different policies. While the latter is the more common formulation in papers on active inference (Friston et al., 2014), we adopt the former here for (graphical) notational convenience. Either would lead to increased direct pathway activation with increased dopamine.

**Figure 8:**
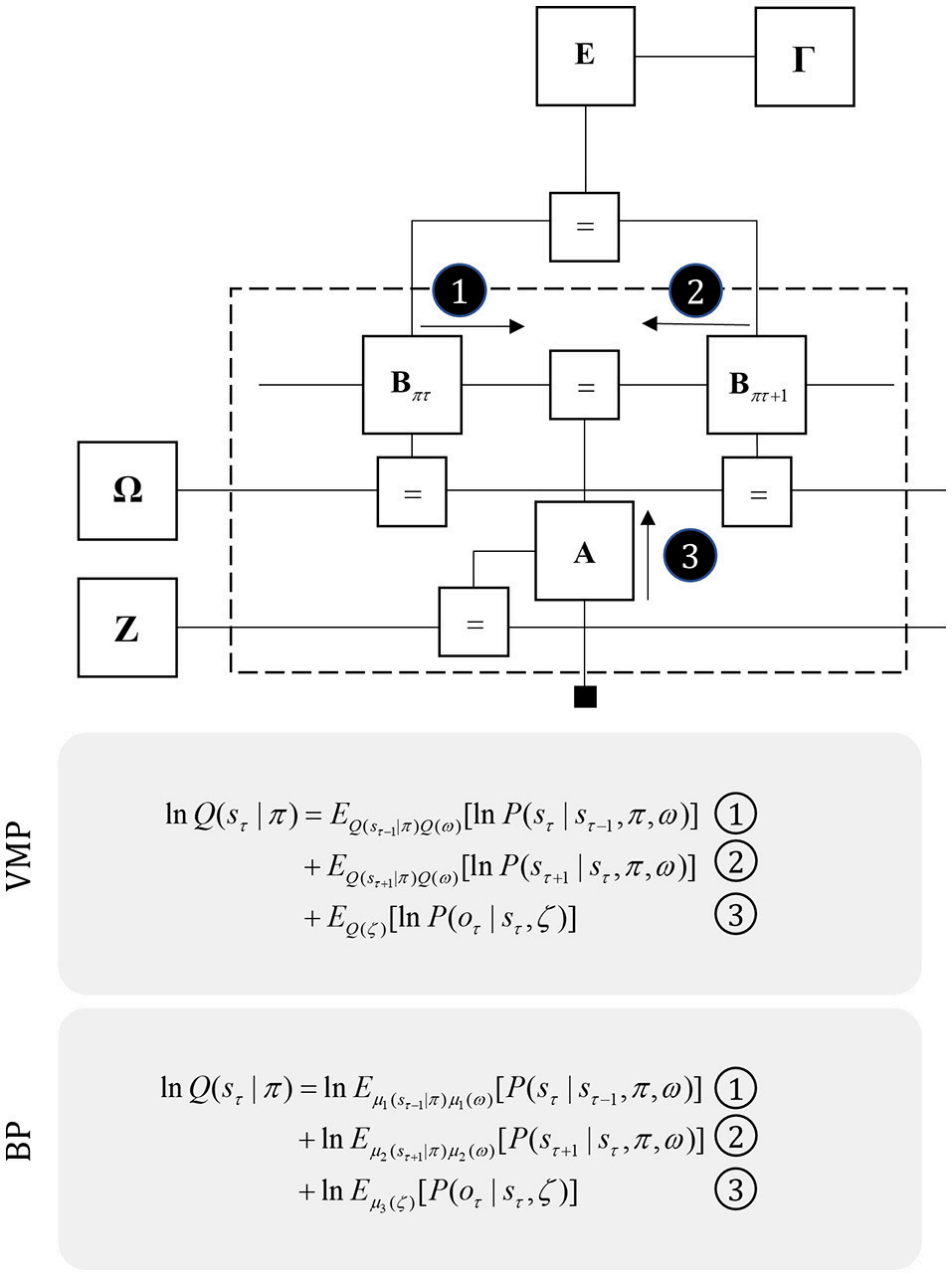
Precision and uncertainty. This figure shows the graph of Figure 5, but supplemented with precision parameters and prior factors over these precisions. These encode confidence in policies (γ), transitions (ω), and likelihoods (ζ). The priors for these are the factors Γ, Ω, and **Z**, respectively. Note that the messages required to update beliefs about the hidden states are almost identical to those of Figure 3, but are now averaged over beliefs about the precision. Messages from the past (1) and future (2) are contextualized by the transition precision, while those from sensory input (3) are modulated by the likelihood precision. As in Figure 3, we provide the form of the variational and belief propagation messages implied by this model to illustrate the commonalities between their forms. Again, this is due to the structure of the Markov blankets of each state, which now includes precision parameters.

We can generalize the idea that dopamine modulates the balance between prior and posterior influences over policies by considering the confidence ascribed to other factors in the generative model. Figure 8 illustrates this idea explicitly by assigning precision parameters to each factor. Prior beliefs about these parameters are expressed in factors Γ, Ω, and **Z**, with precisions (γ, ω, ζ) on the edges connecting to these factors **E**, **B**, and **A** (Parr and Friston, 2017c). The capacity to estimate the precision of these conditional distributions is thought to underwrite attentional processing (Feldman and Friston, 2010). The reason for this is that assigning a high precision (or confidence) to a given probabilistic mapping implies that one variable is highly informative about the other. For example, if we were to hold very precise beliefs about transitions (i.e., a minimally volatile world), the past would be very informative about the present. Similarly, precise beliefs about the likelihood of observing data affords those data the potential to drive belief updating about causative states. In short, increasing precision increases the influence that the messages passed across a factor have on beliefs about variables either side of it. Biologically, this is consistent with modulation of the gain of the synapses carrying these messages. We will use attention, synaptic gain, and precision synonymously in this paper.

### Neuromodulators

**Figure 9:**
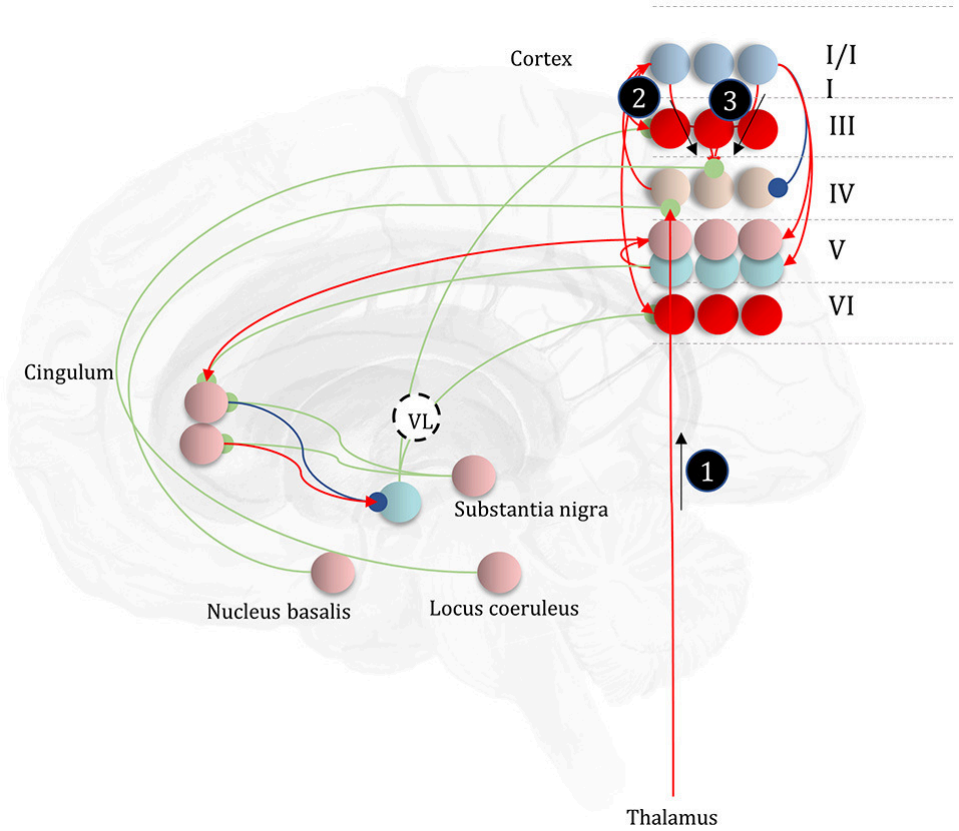
The anatomy of uncertainty. This schematic extends the network of Figure 6 to include modulatory variables, consistent with the factor graph of Figure 8. Specifically, we have now included subcortical regions that give rise to ascending neuromodulatory projections. This includes the locus coeruleus in the pons, which gives rise to noradrenergic signals. Axons from this structure travel through the dorsal noradrenergic bundle to reach the cingulum, a white matter bundle that allows dissemination of signals to much of the cortex. Here, we show these axons modulating messages 2 and 3, representing the past and future, respectively. The nucleus basalis of Meynert is the source of cholinergic signals to the cortex (again, via the cingulum). These modulatory connections target thalamocortical inputs to layer IV (i.e., message 1). Finally, the substantia nigra pars compacta (and the ventral tegmental area) projects via the medial forebrain bundle to the striatum, supplying it with dopaminergic terminals. This modulates the balance between prior and marginal likelihood influences over policy evaluation that we hypothesize correspond to indirect and direct pathway activity, respectively. As before, the layers of the cortical microcircuit shown here represent beliefs about states under a given policy (I/II), beliefs about states averaged over policies (III), state prediction errors (IV), expected free energy gradients and predicted outcomes (V), and beliefs about states averaged over policies (VI).

It is likely that there is a range of mechanisms that give rise to attentional gain-control in the brain, from neuromodulators acting via NMDA receptor pathways (Law-Tho et al., 1993) to communication through coherence (Fries, 2015). While acknowledging that they are only part of the story, we will focus upon the role of ascending neuromodulators (Table 2) in controlling synaptic gain. Figure 9 illustrates the neuronal network implied by the message passing of Figure 8, combined with the network of Figure 6. The additions to this are the subcortical nodes projecting to the cortex. We have associated projections from the locus coeruleus with beliefs about the precision of transitions, consistent with previous theoretical work (Dayan and Yu, 2006; Parr and Friston, 2017c), and with empirical studies (Marshall et al., 2016). The locus coeruleus is the primary source of noradrenaline to the cortex (Aston-Jones and Cohen, 2005), and much of its phenomenology has been reproduced in simulations that associate it with error signals when predicting state transitions (Sales et al., 2018). Error signals of this sort can be interpreted as encoding an increase in the estimated volatility (imprecision) of state transitions.

**Table 2:** Putative roles of neurotransmitters in active inference.

**Neurotransmitter**	**Precision**	**Evidence**
Acetylcholine	Likelihood	•Presence of presynaptic receptors on thalamocortical afferents (Sahin et al., 1992; Lavine et al., 1997) •Modulation of gain of visually evoked responses (Gil et al., 1997; Disney et al., 2007) •Changes in effective connectivity with pharmacological manipulations (Moran et al., 2013) •Modeling of behavioral responses under pharmacological manipulation (Vossel et al., 2014; Marshall et al., 2016)
Noradrenaline	Transitions	•Maintenance of persistent prefrontal (delay-period) activity (requiring precise transition probabilities) depends upon noradrenaline (Arnsten and Li, 2005; Zhang et al., 2013) •Pupillary responses to surprising (i.e., imprecise) sequences (Lavín et al., 2013; Liao et al., 2016; Vincent et al., under review) •Modeling of behavioral responses under pharmacological manipulation (Marshall et al., 2016)
Dopamine	Policies	•Expressed post-synaptically on striatal medium spiny neurons (Freund et al., 1984; Yager et al., 2015) •Computational fMRI reveals midbrain activity with changes in precision (Schwartenbeck et al., 2015) •Modeling of behavioral responses under pharmacological manipulation (Marshall et al., 2016)
Serotonin	Preferences or interoceptive likelihood	•Receptors expressed on layer V pyramidal cells (Aghajanian and Marek, 1999; Lambe et al., 2000; Elliott et al., 2018) in medial prefrontal cortex •Medial prefrontal cortical regions heavily implicated in interoceptive processing and autonomic regulation (Marek et al., 2013; Mukherjee et al., 2016)

The cholinergic system appears a good candidate for the encoding of likelihood precision, given its known role in regulating the gain of sensory evoked responses (Gil et al., 1997; Disney et al., 2007). This implies there should be acetylcholine receptors in those cortical layers receiving messages computed from the likelihood, as in Figure 9. Consistent with this, nicotinic acetylcholine receptors are found presynaptically on thalamocortical terminals targeting layer IV (Sahin et al., 1992; Lavine et al., 1997). Although omitted in Figure 9, the connections labeled “a” and “b” in Figures 5, 6 also depend upon likelihood distributions, and so should be subject to cholinergic input. As the message passing would predict, cholinergic influences are also found in the deeper layers housing these connections (Eckenstein et al., 1988; Arroyo et al., 2014). Both pharmacological (Vossel et al., 2014; Marshall et al., 2016) and neuroimaging (Moran et al., 2013) studies in humans support the hypothesis that the cholinergic system is engaged in precision estimation. This idea additionally lends itself to further testable predictions. A pharmacologically induced decrease in cholinergic activity (e.g., using hyoscine) might then reduce the amplitude of measured electrophysiological responses to sensory stimuli, consistent with increased reliance upon prior beliefs compared to sensory evidence. An attenuated sensory drive toward belief updating might also impair the performance of sensory discrimination tasks (e.g., dot-motion tasks). A further prediction is that a noradrenergic blockade could rescue this performance, as reducing the precision of transition probabilities could restore the relative balance between (empirical) prior and likelihood beliefs.

Neurobiological theories based upon active inference frequently implicate dopamine in the encoding of the precision of beliefs about policies (Friston et al., 2014; FitzGerald et al., 2015). The anatomy of projections originating from the midbrain, compared to the cortex, supports this interpretation. Dopaminergic neurons from the substantia nigra pars compacta and the ventral tegmental area preferentially target the necks of dendritic spines (but also the cell body and axons) of medium spiny neurons (Freund et al., 1984; Yager et al., 2015), while cortical input targets the heads of these, consistent with the notion that dopaminergic signals modulate the gain of these signals rather than providing a driving input. Neuroimaging provides evidence in favor of dopaminergic encoding of precision of beliefs about policies (Schwartenbeck et al., 2015).

While we have focused upon three modulatory transmitters, there are clearly many more to be accounted for in this computational framework (Iglesias et al., 2016; Avery and Krichmar, 2017). One notable omission is serotonin. This transmitter has been linked to various psychiatric conditions, and forms the basis for a range of pharmacological interventions (Andrews et al., 2015). As an illustration of the constraints enforced by the computational anatomy so far, we can use existing knowledge about laminar expressions of serotonin to speculate upon a plausible role. Serotonergic activity is heavily implicated in modulation of layer V pyramidal cells (Aghajanian and Marek, 1999; Lambe et al., 2000; Elliott et al., 2018); especially in the medial prefrontal cortex. Notably, the amygdala receives extensive input from this cell layer (Cho et al., 2013), and cortical region (Marek et al., 2013; Mukherjee et al., 2016).

Drawing from the idea that some nuclei of the amygdala could be an autonomic analog of the basal ganglia (Swanson and Petrovich, 1998) (see the section Planning for details), we can hypothesize that serotonin is somehow involved in modulating policy selection in response to interoceptive signals. The involvement of layer V suggests two mechanisms by which this might occur. The inputs to this layer in Figure 6 include those that depend upon the likelihood (labeled “a” and “b”) and descending messages representing the top-down influence via the **C** factor (i.e., context sensitive preferences); i.e., message 3 in Figure 6. Notably, both of these are functions of predicted outcomes, which in this case would be interoceptive modalities. This suggests two alternative hypotheses for the computational role of serotonin. Either it plays an analogous role to ζ, the likelihood precision (i.e., an interoceptive version of acetylcholine), or it could modulate an equivalent precision parameter encoding the fidelity of the mapping from high level states (context) to interoceptive preferences. Either of these hypotheses complement the recent trend toward embodied psychiatry (Seth, 2013; Barrett et al., 2016; Petzschner et al., 2017; Khalsa et al., 2018), and longstanding theories concerning the connection between mood and interoceptive sensations (James, 1884; Ondobaka et al., 2017).

### Inferring uncertainty

For simplicity, we have only included the unidirectional connections from neuromodulatory systems to the cortex and basal ganglia in Figure 9. The form of Figure 8 demonstrates that the Markov blankets of these precision parameters include the variables encoded by the pre and postsynaptic neurons of the synapses they modulate. For the two cortically-projecting systems, this implicates axons signaling in the reverse direction in the cingulum, perhaps targeting the prefrontal cortex (which projects to both the nucleus basalis and the locus coeruleus). For the mesostriatal system, this suggests reciprocal interactions between the dopaminergic midbrain and the striatum, consistent with known anatomical loops between these structures (Haber, 2003), and the striosome compartments of the striatum that seem specialized in relaying signals to the dopaminergic midbrain (Fujiyama et al., 2011).

### Pathologies of synaptic gain

There are many disorders thought to be due to abnormalities of precision estimation (Friston, 2017; Friston et al., 2017b; Parr et al., 2018b) and synaptic gain, including but not limited to Lewy body dementia, Autism, and Parkinson's disease. Theoretical accounts of the first of these typically implicate abnormalities in estimating likelihood precision (Collerton et al., 2005; Parr et al., 2018a). Recent accounts of autism suggest a failure to properly estimate the precision of transitions (Lawson et al., 2014, 2017). As discussed in the section Planning, Parkinson's disease reflects degeneration of the dopaminergic system, leading to failure to represent the precision of beliefs about policies. The above conditions show changes in neurotransmitter function consistent with the computational anatomy of Figure 9. In Lewy body dementia, there is a dramatic decrease in cholinergic activity in the cortex (Perry et al., 1994; Graff-Radford et al., 2012), effectively releasing the cortex from the constraints imposed by sensory input (message 1). This could account for the complex visual hallucinations associated with this condition. Pupillary analysis in autism indicates attenuated responses to (normally) surprising stimuli (Lawson et al., 2017). Given the relationship between pupillary dilatation and the activity of the locus coeruleus (Koss, 1986), this implies abnormalities in noradrenergic signaling, limiting the influence that beliefs about the past (message 2) and the future (message 3). This could lead to an excessive reliance upon message 1, and a failure to use prior beliefs to contextualize this sensory evidence. Theoretical accounts of this sort (Palmer et al., 2015, 2017) have been used to account for the resistance to visual illusions (Happé, 1996) and the superior visual search performance observed in autistic individuals (Shah and Frith, 1983; Simmons et al., 2009).

## Movement

### Predictions and motor commands

The graphs and neuronal networks of the preceding sections have all focused upon the discrete dynamics outlined on the left of Figure 3. While this is appropriate for planning sequences of actions, these models are not suited to implementing these actions as movements. The reason for this is that movement necessarily involves continuous variables (muscle length, velocity) that evolve in continuous time, as indicated on the right of Figure 3 (Parr and Friston, 2018b). The same is true of low-level sensory processes, as data arrives in continuous time. Fortunately, the structure of the message passing is almost identical in the two cases (Friston et al., 2017a). Again, they correspond to reciprocal connections between neurons representing prediction errors (free energy gradients) and posterior expectations [for more details on this, please see (Bastos et al., 2012; Shipp, 2016)]. This means we can use the same sorts of architectures to make predictions about the continuous signals from muscle proprioceptors. Active inference takes this one step further. Once we have a prediction as to the sensory data coming from a muscle, this generates a prediction error. There are two ways in which this prediction error may be resolved. First, we could update expectations to be more consistent with this. Second, we could use the error to drive a movement that renders proprioceptive data consistent with the prediction (Adams et al., 2013). Under this view, motor commands and proprioceptive predictions become synonymous. Brainstem and spinal cord reflexes can then be seen as mechanisms to resolve prediction errors. An interesting consequence of this is that, given errors are resolved at this level, there should be no residual error at the level of the motor cortex, as all of its expectations are fulfilled at spinal levels. As such, cells in the motor cortex representing prediction error may be redundant. This reasoning has been used to account for the impoverished (granular) layer IV in primary motor cortex (Shipp et al., 2013), sometimes referred to as “agranular” cortex for this reason. However, it seems that some prediction error must still be unresolved, as there is evidence for some granular cells in motor cortex (García-Cabezas and Barbas, 2014; Barbas and García-Cabezas, 2015).

In addition to accounting for anatomical findings, models based upon this form of active inference have reproduced a range of complex motor phenomena, including handwriting (Friston et al., 2011), limb movements (Friston et al., 2010), smooth pursuit (Adams et al., 2012), and saccadic eye movements (Friston et al., 2012a). They are capable of reproducing plausible electrophysiological and pathological behaviors that are consistent with (clinical) neuroanatomy. For example, we have previously reproduced the activity of collicular “build-up” cells (Ma et al., 1991; Munoz and Wurtz, 1995) and pathological phenomena, including internuclear ophthalmoplegia (Virgo and Plant, 2017), using the same generative model (Parr and Friston, 2018a).

### Translating policies into movements

**Figure 10:**
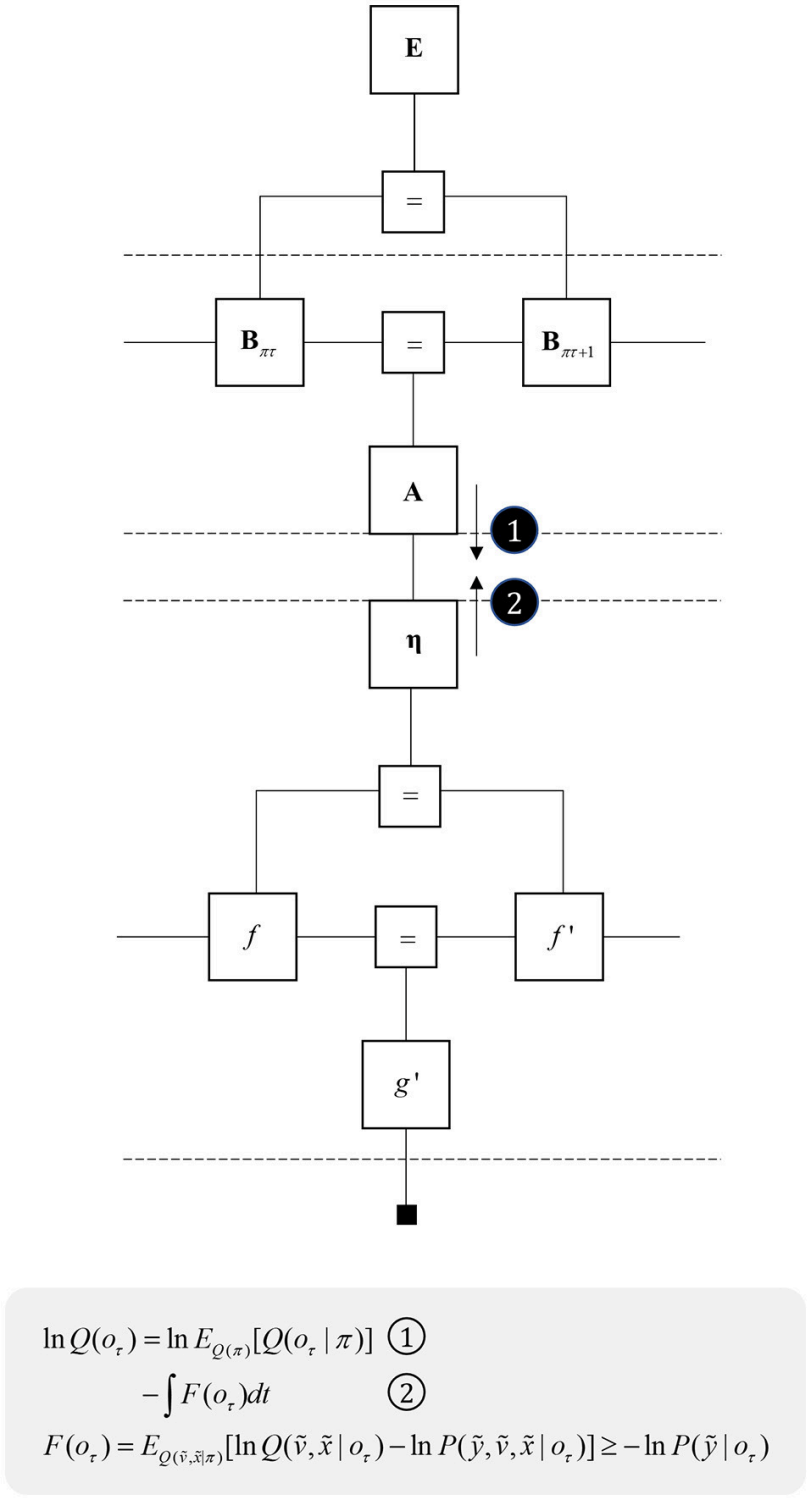
Decisions and movement. This graph illustrates how beliefs about categorical variables may influence those about continuous variables (message 1) and vice versa (message 2). The upper part of the graph is the same as that from Figure 5, while the lower part is that from the right of Figure 2. The additional η factor represents the empirical prior for a hidden cause, *v*, that determines the dynamics of *x*, much as the policies at the higher level determine the dynamics of state transitions. The equations below show that we can treat the descending message as a Bayesian model average, incorporating posterior predictive beliefs about outcomes under policies, averaged over policies. The ascending message is the free energy integrated over time for each outcome. This effectively treats each outcome as an alternative hypothesis for the continuous dynamics at the lower level.

The success of continuous state space generative models in accounting for motor behavior appears to imply a disconnect between movement and planning, with the latter more easily accounted for using discrete time models. This suggests there must be some interface between the two, where decisions, selected from a discrete repertoire, are translated into beliefs about continuous variables. Figure 10 illustrates this idea, with a discrete model that gives rise to empirical priors for a continuous model, via a η factor. This corresponds to a Bayesian model average, where several hypothetical continuous variables are weighted by the probabilities of their associated categorical outcomes (Friston et al., 2017a). By computing the approximate partition function (negative free energy) for the continuous region, we can approximate the evidence for each categorical outcome. As the continuous dynamics play out over time, the log evidence must be integrated over that duration. The idea that complex motor behavior may be constructed from short sequences of simpler dynamics resonates with ideas implemented in control systems and robotics (Schaal, 2006).

### Discretized encoding of continuous space

**Figure 11:**
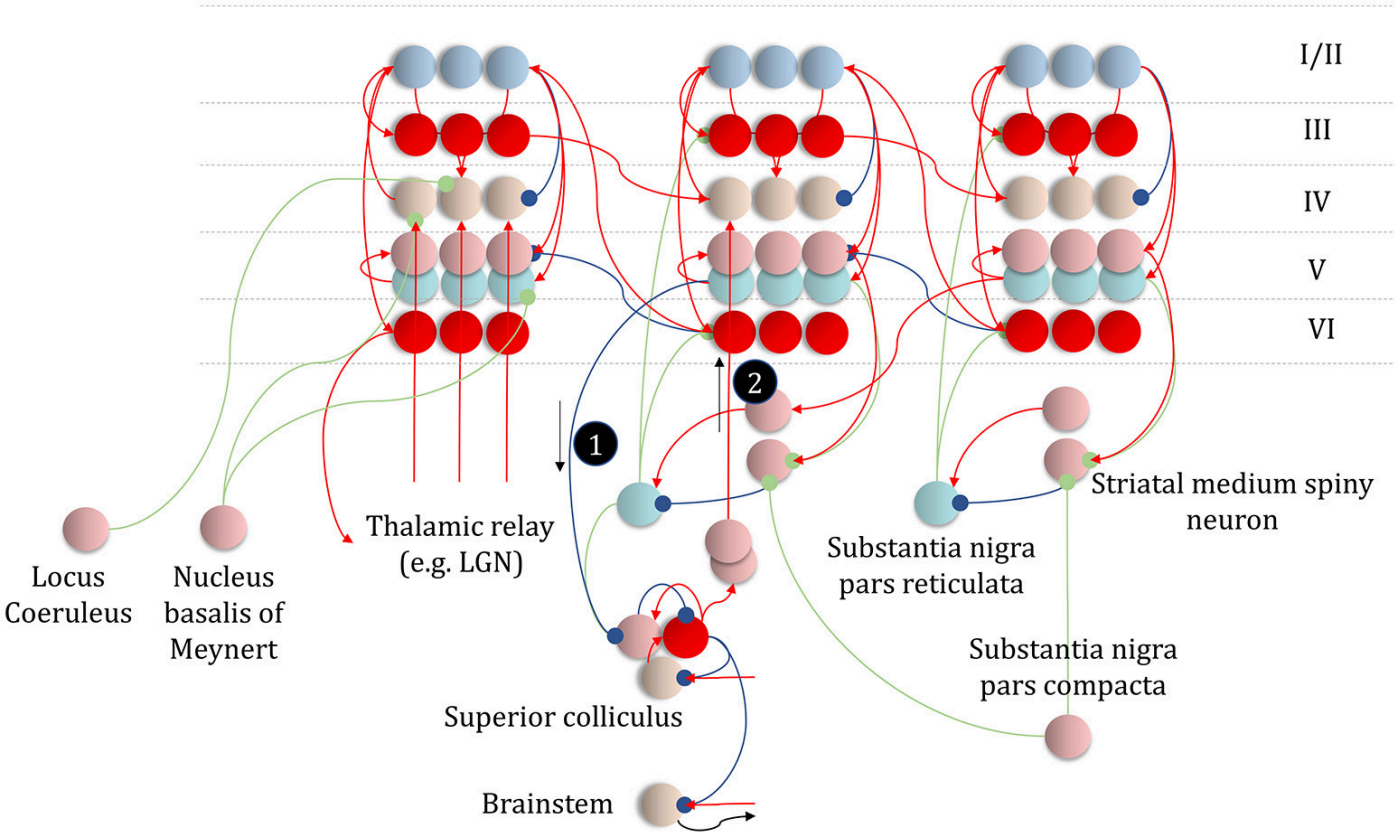
An anatomy of inference. This schematic summarizes the networks we have discussed so far, but adds in the messages of Figure 10, with empirical priors propagated by message 1. These are subtracted from expectation neurons to give error signals, then used to update expectations. Expectations are used to derive predictions about sensory data. These are subtracted from the incoming data to calculate sensory errors, used to update current expectations, but also to drive brainstem reflexes through action (black arrow) to change sensory data (e.g., by moving the eyes). Message 2 derives from the expectations, which are used to compute the integral of the free energy over time. The relative evidence for each outcome is then propagated to layer IV cells in the cortex, acting as if it were sensory data. As before, the layers of the cortical microcircuit shown here represent beliefs about states under a given policy (I/II), beliefs about states averaged over policies (III), state prediction errors (IV), expected free energy gradients and predicted outcomes (V), and beliefs about states averaged over policies (VI).

Translating from discrete to continuous variables implies that there must be an interface at which a discretized encoding of space is mapped to a continuous encoding. In the oculomotor system, the superior colliculus may represent an interface of this sort (Parr and Friston, 2018b). It contains a population of cells that are retinotopically mapped to regions of visual space (Sparks, 1986), but the brainstem oculomotor regions it projects to appear to encode continuous variables. It is ideally placed to map empirical priors, derived from cortical beliefs, to predictions about eye position and the status of oculomotor muscles. Its cortical input is derived from cells in layer V (Fries, 1985; Kim et al., 2015), possibly those encoding posterior predictive beliefs about discrete outcomes under each policy (Figure 6). The superior colliculus additionally receives input from the substantia nigra pars reticulata (Hikosaka and Wurtz, 1983), which could encode posterior beliefs about policies in line with Figure 6. This means the superior colliculus receives the inputs required to perform a Bayesian model average over policies to derive empirical priors over outcomes, and over the causes of continuous dynamics (message 1 in Figures 10, 11).

While the superior colliculus may play the role of discrete-continuous interface in the oculomotor system, other structures must play analogous roles for different motor outputs. These are likely to share features of anatomy and physiology with the colliculus. Following the pattern above, these structures should receive input from cortical layer V, and from the output nuclei of the basal ganglia. Furthermore, they should encode continuous variables in a discretized fashion, with different neurons representing discrete elements of a continuous scale. A network that includes the ventrolateral (motor) thalamus and the primary motor cortex represent a candidate that meets these criteria (Bosch-Bouju et al., 2013). The motor thalamus receives cortical layer V and basal ganglia input, and projects to motor cortical regions. This suggests the combined cortico-subcortical input to these thalamic nuclei could represent message 1 in Figure 10. Thalamic projections to primary motor cortex might then be the axonal substrate of the η factor. The motor cortex is known to contain discretized maps of space (Georgopoulos et al., 1986), while the spinal motor neurons it projects to elicit continuous changes in muscle length, depending upon their firing rates (Connelly et al., 1999; Conwit et al., 1999; Kirk and Rice, 2017). This implies the motor thalamus and motor cortex might together play the same role for limb and trunk movements as the subpopulations within the superior colliculus do for eye movements.

Figure 11 places these ideas in the context of the neuronal networks from previous sections, showing a hierarchy of three cortical areas, one of which gives rise to projections to the superior colliculus. This would be consistent with a hierarchy implicating occipito-parietal areas at the lowest level (left column) that project to, and receive projections from, frontal oculomotor areas (middle) known to project to the superior colliculus (Künzle and Akert, 1977). These then share reciprocal connections with dorsolateral parts of the prefrontal cortex, involved in the longer-term planning of eye movements required for delayed oculomotor responses (Fuster et al., 1982; Funahashi et al., 1989). The laminar specific terminations in Figure 11 conform to those required for message passing in the generative models we have described, and are highly consistent with those observed in the cerebral cortex and associated structures (Shipp, 2007).

## Discussion

The preceding sections have reviewed recent attempts to understand the anatomy of the brain in terms of the inferential computations it must perform. We have argued that the key determinant for anatomical connectivity is the structure of the generative model the brain uses to make these inferences. This allows us to express hypotheses about computational neuroanatomy in a graphical notation that can be built up from relatively few simple building blocks, as described above. This framework is sufficiently general that we can use it to understand perceptual inference, planning, attentional gain, and movement. These can all be combined within the same factor graph, enabling the expression of systems-level hypotheses about brain-wide networks.

This article has focused on some very specific but ubiquitous features of computational anatomy that emerge under a factor graph treatment—with special attention to known neuroanatomy, neurophysiology, and neuropsychology. There are clear and obvious architectural features that are predicted under a graphical treatment of neuronal message passing; for example, the very existence of sparse neuronal (axonal) connections and the hierarchical organization of cortical and subcortical structures. The very existence of the brain as a network or graph that possesses hierarchically nested Markov blankets—and engages in sparse message passing [unlike the liver or blood (Friston and Buzsaki, 2016)]—could be understood as a prediction of the process theories that arise under active inference and Bayesian brain. Crucially, the formal approach offered by these process theories forces us to ensure consistency in theories about different aspects of brain function. For example, the assignment of posterior predictive beliefs and expected free energy gradients to cortical layer V in the section on Planning had to be consistent with the kinds of signals propagated to the superior colliculus, and the motor thalamus, from this same cortical layer in the section on Movement. This represents one of many constraints that can be simply articulated using the graphical formalisms described here.

Not only do these ideas have to be internally consistent (minimally complex in relation to one another), they must accurately account for a range of observed phenomena, including the consequences of anatomical lesions. We have outlined a few examples throughout that illustrate this, including abnormalities of perception resulting from disconnections (e.g., Charles Bonnet syndrome), disorders of policy evaluation (e.g., Parkinson's disease), and failures of attentional gain (e.g., Lewy body disease). It is also important to realize that, as messages are propagated across the graph, deficits in one part of the graph have implications for all other parts. A disorder that offers a clear example of this kind of diaschisis (Price et al., 2001; Carrera and Tononi, 2014) is visual neglect (Parr and Friston, 2017a). This neuropsychological syndrome is associated with right hemispheric lesions (Halligan and Marshall, 1998), which can occur at various anatomical sites, and results in a failure to perform exploratory saccades to the left side of visual space (Husain et al., 2001; Fruhmann Berger et al., 2008; Karnath and Rorden, 2012).

The heterogeneity of anatomical lesions giving rise to neglect illustrates that the same processes of policy (i.e., saccadic) selection can be disrupted by multiple distant lesions. We have previously shown through simulation (Parr and Friston, 2017b) that disruption of policy priors (**E**), proprioceptive preferences (**C**), or the likelihood mapping fixation locations to predicted visual data (**A**) can all bias saccadic policy selection. This is unsurprising when we consider the factor graph of Figure 5, as messages across each of these factors either directly or indirectly influences beliefs about policies. Lesions in the proposed neurobiological substrates (Parr et al., 2018b) of each of these factors have been associated with visual neglect (Karnath et al., 2002; Bartolomeo, 2012). Although not commonly observed in clinical practice, experimental manipulation of almost every part of the anatomy presented here can induce or alleviate neglect-like saccadic behavior, including unilateral collicular inactivation (Schiller et al., 1980, 1987), chemical ablation of the substantia nigra pars compacta (Kato et al., 1995; Kori et al., 1995), and noradrenergic modulation (Malhotra et al., 2006).

The above represent criteria for the face validity of anatomical process theories. To go further, it is necessary to make empirical predictions based upon these theories. We have highlighted three novel ideas that have arisen from the form of the generative models used here, which could be interrogated in empirical studies. First, if we interpret the direct and indirect pathways of the basal ganglia in terms of partition functions and empirical priors, respectively, this has important consequences for learned behaviors. While it is possible to optimize the parameters of a conditional probability (**E**), the same cannot be done for the partition function; although it is possible to optimize those distributions that make up that function. This suggests that learned automatic behaviors depend upon plastic changes involving the indirect, more than the direct, pathway. Selective ablation or optogenetic suppression (Freeze et al., 2013) of the direct pathway would, under this hypothesis, preserve certain context dependent automatic behaviors. In other words, it should be possible to reproduce the phenomenon of kinesia paradoxa by facilitating the indirect at the expense of the direct pathway, perhaps while presenting slowly changing exteroceptive cues with a learned behavioral association.

Second, we touched upon hypothetical computational roles for serotonin that would be consistent with its anatomical and laminar distribution in the cortex under the computational anatomy discussed above. This scheme offers the potential to frame these, anatomically derived, computational hypotheses in terms of simulated behavior. To test whether serotonergic modulations are best explained as manipulations of interoceptive sensory precision, or the precision of preferences, we would need to design a task in which simulating manipulations of each of these parameters would give rise to different behavioral outputs. Fitting these parameters to behavior under different levels of pharmacological manipulation would allow us to evaluate the relative evidence for each of these hypotheses. For a recent example of this sort of approach, inferring computational parameters (including precision of preferences) for visual exploration, see Mirza et al. (2018).

Finally, we considered the role of the motor thalamocortical networks, and suggested that these might represent the sort of discrete-continuous interface that we have previously associated with the superior colliculus. This predicts that there should be a very different sort of deficit resulting from the pathway into the ventrolateral thalamus compared to that following lesions to motor cortical outputs. The former might involve deficits in choice of movement, or difficulty initiating movements. The latter are more likely to give rise to impairments in the motor trajectories themselves. Of course, it is important to emphasize again that lesions to any neuroanatomical structure, or equivalently, to any part of a generative model, will have wide-reaching consequences due to the propagation of inferential messages.

The above represent theoretically motivated hypotheses that may be evaluated in relation to empirical evidence. These are potentially falsifiable (in a frequentist statistical sense), or could be shown to be relatively poor hypotheses compared to an alternative explanation (in a Bayesian sense). It is worth emphasizing that the inferential framework described here is not subject to these same tests. This (active) inference formulation simply provides a formal language and notation in which hypotheses about neuronal processes can be articulated and evaluated. The formulation of the brain's inferential computations as graphs and Markov blankets is therefore not in competition with, or an endorsement of, other approaches to understanding brain function. It accommodates those approaches that appeal to chaotic dynamical systems (Korn and Faure, 2003), as these may be written in to the flows of Figures 3, 10 (Friston and Ao, 2012), and is predicated upon probabilistic dynamics of the sort that motivate the use of mathematical tools developed in stochastic (and quantum) physics (Seifert, 2012; Korf, 2014) for understanding brain function.

Clearly the account given in this paper is far from complete. We have omitted important structures from this, including the cerebellum and second order thalamic nuclei, like the pulvinar. These have not escaped treatment under the framework of active inference. The pulvinar, have been associated with generative models that treat prior beliefs over precisions as empirical priors, conditioned upon hidden states (Kanai et al., 2015). This sort of state-dependent precision is vital in accounting for phenomena such as figure-ground segregation. The cerebellum has been associated with inferences and learning in the continuous domain (Friston and Herreros, 2016), enabling Pavlovian conditioning of eye-blink responses. For an account of the cerebellum consistent with our computational anatomy, it is worth noting that the cerebellum projects to both the ventrolateral nucleus of the thalamus, and to the superior colliculus. These are the regions we have associated with discrete-continuous interfaces, and therefore with the η factor of Figure 10. This raises the possibility that one of the roles of the cerebellum is to optimize the mapping between discrete motor sequences, and the trajectories at each point in the sequence. This resonates with features of cerebellar disease, including “past-pointing,” where patients are able to recognize a target and initiate a limb movement toward it, but fail to map the location of the target effectively into continuous space, and miss when trying to point to it. Future work developing the anatomical process theory of active inference must rise to the challenge of synthesizing these phenomena within this broader theory.

## Conclusion

In this paper, we have emphasized the idea that generative models, and their constituent Markov blankets, represent a useful way to express hypotheses about brain connectivity. We have reviewed recent attempts to apply this framework to a range of anatomical networks, illustrating their face validity and internal consistency. There may be other plausible mappings between the connectivity implied by the Markov blankets of a generative model and the anatomy of the brain, which could make use of different auxiliary variables to the free energy gradients (prediction errors) we have assumed. Similarly, there are other plausible generative models that the brain may use, and these may involve different Markov blankets. For this reason, we emphasize not only current anatomical theories, but also a theoretically rigorous graphical framework in which questions about computational anatomy can be clearly posed. Under this framework, there are two broad lines of enquiry. First, what are the generative models the brain employs to make inferences about the world? Second, what is the mapping between the network implied by a given generative model and the connections of the brain? These questions constrain one another, as a good hypothesis for a computational neuroanatomy will imply a plausible generative model that contains Markov blankets consistent with brain connectivity.

## Author contributions

All authors listed have made a substantial, direct and intellectual contribution to the work, and approved it for publication.

### Conflict of interest statement

The authors declare that the research was conducted in the absence of any commercial or financial relationships that could be construed as a potential conflict of interest.

## Footnotes

^1^An empirical prior arises in hierarchical models, when a higher level provides constraints on a lower-level. It is called an empirical prior because the reciprocal message passing means that the prior is informed by the (empirical) data at hand. In other words, hierarchical models endow (empirical) priors with context-sensitivity.

^2^The (Shannon) entropy is the dispersion of a probability distribution, and may be thought of as quantifying uncertainty about the variable described by the distribution.
