# Supplementary material for: The Anatomy of Inference: Generative Models and Brain Structure
Source: Front Comput Neurosci. 2018 Nov 13;12:90. doi: 10.3389/fncom.2018.00090 (PMC6243103; doi:10.3389/fncom.2018.00090)
Supplement: Supplementary file 1 [file Data_Sheet_1.pdf]

Appendix

A.1 – Bayesian message passing

This appendix provides a brief technical overview of two Bayesian message passing schemes referred to in the main text. These are belief propagation and variational message passing – each of which use locally derived information (i.e. from a variable's Markov blanket) to compute beliefs (Q) about that variable. Belief propagation relies upon a set of recursive updates that ultimately express a posterior belief as the product of messages μ from neighbouring factors. For example, the posterior obtained using this approach for the graph of Figure 3 gives:

$$
\begin{aligned}
&Q(s_\tau) \propto \overrightarrow{\mu}_B(s_\tau) \cdot \mu_A(s_\tau) \cdot \overleftarrow{\mu}_B(s_\tau)\\
&\overrightarrow{\mu}_B(s_\tau) = E_{\overrightarrow{\mu}_B(s_{\tau-1})\mu_A(s_{\tau-1})}[P(s_\tau \mid s_{\tau-1})]\\
&\mu_A(s_\tau) = P(o_\tau \mid s_\tau)\\
&\overleftarrow{\mu}_B(s_\tau) = E_{\overleftarrow{\mu}_B(s_{\tau+1})\mu_A(s_{\tau+1})}[P(s_{\tau+1} \mid s_\tau)]
\end{aligned}
$$

Here, the notation $E[\ \ ]$ means the 'expectation' of the term within the square brackets with respect to the subscripted distribution. Note that the messages here are defined in terms of other messages.

Similarly, variational message passing may be written as a product of messages, but with a subtly different form:

$$
\begin{aligned}
&Q(s_\tau) \propto \overrightarrow{\nu}_B(s_\tau) \cdot \nu_A(s_\tau) \cdot \overleftarrow{\nu}_B(s_\tau)\\
&\overrightarrow{\nu}_B(s_\tau) = \exp(E_{Q(s_{\tau-1})}[\ln P(s_\tau \mid s_{\tau-1})])\\
&\nu_A(s_\tau) = P(o_\tau \mid s_\tau)\\
&\overleftarrow{\nu}_B(s_\tau) = \exp(E_{Q(s_{\tau+1})}[\ln P(s_{\tau+1} \mid s_\tau)])
\end{aligned}
$$

The expectations here are with respect to beliefs, as opposed to the messages of belief propagation, avoiding the need to resort to a recursion. However, the key point here is the similarity in the form of these two schemes. Each relies upon the structure of the Markov blanket, and uses only locally available information to compute the posterior.

A.2 – Free energy and partition functions

This appendix unpacks the use of free energy functionals (functions of functions) as approximations of partition functions, and emphasises how an expected free energy may be interpreted in terms of explorative and exploitative drives. Using the model shown in Figure 5, we might hope, by 'closing the box', to compute the evidence for a policy:

$$
\ln P(o_\tau \mid \pi) = \ln \sum_{s_\tau} P(o_\tau, s_\tau \mid \pi)
$$

This summation (or integral for a continuous state-space model) is often difficult to perform analytically, so we can instead rely upon Jensen's inequality ('the log of an average is greater than or equal to the average of a log') to approximate this with a free energy functional:

$$
\begin{aligned}
&\ln P(o_\tau \mid \pi) \geq -F(\pi,\tau) \\
&F(\pi,\tau) = E_{Q(s_\tau|\pi)}[\ln Q(s_\tau \mid \pi) - \ln P(o_\tau, s_\tau \mid \pi)]
\end{aligned}
$$

This provides a lower bound upon the log evidence (or partition function) for a given policy. The *Q* that optimises this bound (i.e. leads to the best approximation of the log evidence by the negative free energy) is that given by the message passing schemes described in A.1.

To compute the approximate partition function for yet-to-be-observed data (i.e. future trajectories), we need to find the free energy under predicted outcomes. We can express predicted outcomes, based upon current beliefs, as:

$$
\tilde{Q}(o_\tau, s_\tau \mid \pi) = P(o_\tau \mid s_\tau)Q(s_\tau \mid \pi)
$$

This allows us to define an expected free energy:

$$
\begin{aligned}
G(\pi,\tau) &= E_{\tilde{Q}(o_\tau, s_\tau|\pi)}[\ln Q(s_\tau \mid \pi) - \ln P(o_\tau, s_\tau)] \\
&= E_{Q(s_\tau|\pi)}[\underbrace{H[P(o_\tau \mid s_\tau)]}_{Ambiguity}] + \underbrace{D_{KL}[Q(o_\tau \mid \pi) \,\|\, P(o_\tau)]}_{Risk}
\end{aligned}
$$

The second line here indicates that those policies with the smallest expected free energy are those that lead to unambiguous (minimally uncertain) state-outcome mappings, and which lead to outcomes consistent with prior beliefs about observations. As such, those policies that are associated with a high expected (approximate) evidence will involve exploration (information gain) and exploitation (fulfilment of prior preferences).
